# Biochemical Reference Intervals of Free‐Ranging Koalas ( *Phascolarctos cinereus* ) in South Australia

**DOI:** 10.1111/vcp.70024

**Published:** 2025-07-02

**Authors:** María B. Font, Lucy Woolford, Sue Jaensch, Doug Hayward, Michelle Hebart, Emily Dean, Wayne S. J. Boardman, Tamsyn Stephenson, Jessica Kovac, Natasha Speight

**Affiliations:** ^1^ Faculty of Exact and Natural Sciences, University of Buenos Aires Buenos Aires Argentina; ^2^ School of Animal and Veterinary Sciences Faculty of Science, Engineering and Technology, University of Adelaide Adelaide South Australia Australia; ^3^ Vetnostics Laboratory North Ryde New South Wales Australia

**Keywords:** *Chlamydia*, health, koala retrovirus, Phascolarctidae, reference intervals, serum

## Abstract

**Background:**

Reference intervals (RIs) are an essential tool for assessment of clinical pathology data of animals, and are particularly important for monitoring the health status of free‐ranging and captive wildlife, such as koalas ( *Phascolarctos cinereus* ).

**Objectives:**

The purpose of this study was to: (1) provide comprehensive serum biochemistry reference intervals based on clinically healthy South Australian koalas from two populations, Mount Lofty Ranges (MLR) and Kangaroo Island (KI); and (2) identify any factors that can affect biochemical analytes, including koala retrovirus (KoRV) and *Chlamydia pecorum* subclinical infection status, age, sex, and population.

**Methods:**

Serum biochemistry analytes were determined in 206 clinically healthy South Australian koalas caught from the wild in 2016 and 2018 using a Cobas 8000 Chemistry Analyzer and analyzed using Reference Value Advisor and SPSS v28 Statistical software.

**Results:**

Biochemical reference intervals were established. Also, clinically and statistically significant differences in analytes were found based on age for alkaline phosphatase and phosphate, and albumin: globulin ratio, globulins, and total protein, most likely associated with physiological bone growth and immunological development, respectively, as observed in other species. Statistically significant differences between animals subclinically positive for KoRV and *Chlamydia pecorum* , were found for glucose and gamma glutamyl transferase respectively; however, these were marginal, and their reference intervals were similar.

**Conclusions:**

This study is the first to describe serum biochemical reference intervals for clinically healthy South Australian koalas of known *Chlamydia* and KoRV infection status. It represents an important tool to assist health assessments of koalas by veterinarians, as well as research and population monitoring.

## 1 Introduction

The koala ( *Phascolarctos cinereus* ) is a medium‐sized, folivorous arboreal marsupial [1], and is the only extant species in the family Phascolarctidae. Its distribution corresponds to the east coast of Australia, with a fragmented distribution across its habitat of *Eucalyptus* spp. forest [1]. Since the beginning of the 20th century, the natural range of the koala has declined dramatically, initially due to European settlement, followed by habitat loss, urbanization, drought, bushfires, and diseases, such as *Chlamydia pecorum* [2]. The Australian federal government has recently reclassified koalas from “vulnerable” to “endangered” in most of their range (NSW, ACT, and QLD) [3].

In South Australia (SA), the two main populations are the mainland Mount Lofty Ranges (MLR) population and the Kangaroo Island (KI) population. Their health status has been investigated by a number of recent studies, identifying several key threats such as koala retrovirus (KoRV), *Chlamydia pecorum*, and oxalate nephrosis. KoRV is a pathogen that has been found at lower prevalence and loads in free‐ranging populations of koalas in SA compared with northern koalas, with rates of 42.4% in KI and 65.3% in MLR [4]. KoRV may predispose to periodontal diseases (PD), such as gingivitis and periodontitis, lymphoid neoplasia, or even concomitant disease with *Chlamydia*, due to immunological suppression, but may also be subclinical [5, 6, 7]. In SA, MLR koalas show fragmented expression of KoRV, which may indicate dysfunction and lack of clinical consequences [8]. Overt chlamydial disease occurs at low levels in SA koalas, whereby 46.7% of wild‐caught MLR koalas were found to be subclinically infected ( *C. pecorum* positive) by quantitative PCR, but only 4% presented overt clinical disease [9]. Furthermore, the KI koalas may be one of the only large populations of *C. pecorum* ‐free koalas in Australia, with 95% confidence [9]. Oxalate nephrosis, however, is a common cause of morbidity and mortality in the MLR population, affecting 32% of rescued wild koalas and causing renal dysfunction [10, 11, 12]. Accurate diagnostic tests are therefore required for koalas undergoing clinical examination to determine their health status.

Haematologic and biochemical analyses are an integral component of disease diagnosis and clinical decision‐making in domestic animals. Ideally, a reference interval (RI) should be established for each animal population, and the reference population should represent the animal for which the RI will be used [13]. In the case of koalas, there have been previous studies of haematologic and biochemical values [14, 15, 16, 17, 18]. A 1975 study by Dickens [15] reported biochemistry ranges for more than 200 koalas from Queensland, New South Wales, and Victoria; however, RIs were not calculated. Following this, a 1989 study calculated biochemical reference values based on a small number of koalas (*n* = 45) of apparent good health from New South Wales, of uncertain *C. pecorum* and KoRV status [18].

Thus, the aim of this study was to develop comprehensive serum biochemistry RIs based on 206 clinically healthy free‐ranging koalas from two SA populations, MLR and KI, sampled between 2016 and 2018, and to determine if there are significant differences in these values based on sex, age, and population, with known KoRV and *C. pecorum* subclinical infection status. The RIs generated in this study are intended to improve the assessment of the health of wild and captive koalas, a benefit to both veterinary medicine and koala research communities.

## 2 Materials and Methods

Blood samples were collected from 230 koalas from two wild populations in South Australia over 1 week periods between 2016 and 2018 as part of studies to assess wild koalas' health. For examination, koalas from MLR were anesthetized with injectable agents (alfaxalone and medetomidine) [19] and koalas from KI were anesthetized with isoflurane inhalation anesthesia and oxygen mask. Health was assessed by veterinarians experienced in koala health through a combination of factors, including sex, age, body condition score, coat and skin condition, and external clinical findings. Age was assessed by dentition using the Tooth Wear Class classification (TWC; TWC I = 1–2 years; TWC II = 2–3 years; TWC III = 4 years; TWC IV = 4–9 years, TWC V = 10–12 years, TWC VI = 12+ years) [1] and koalas were further categorized into age groups: independent juvenile (J, TWC I), young adult (YA, TWC II‐III), and adult (A, TWC > IV). Koala body condition score (BCS) was estimated by the scapular musculature as 1–5 (poor to excellent) [20], and modified to a 3‐point scale of poor, good and excellent (where poor < 3, good = 3, excellent > 3 out of 5).

Blood was collected from the cephalic or femoral vein into plain tubes, and serum refrigerated until freezing within 24 h at −20°C, prior to storage at −80°C. Validation of freezing koala serum for biochemical analysis has not been previously investigated, including for long storage periods (4 to 6 years in the current study), however studies in other animals show minimal changes over a period of 1 year [21].

Of the 230 koalas, only samples from 206 koalas (MLR April 2016 *n* = 52, and 2018 *n* = 61 [total MLR = 113] and KI February 2017 *n* = 93) progressed to data analysis due to the study exclusion criteria, whereby only those animals without clinical diseases or injuries, and with good or excellent body condition, were included in the study. Thus, the final number of samples (*n* = 206) excluded koalas with clinical signs of *C. pecorum* (*n* = 9) and those with a poor body condition score (BCS) (*n* = 15). The koalas from KI were females only, since the samples were collected alongside the state government's Department of Environment and Water (DEW) koala management program. The sampling of wild koalas was approved by the University of Adelaide Animal Ethics Committee, S‐2013‐198, S‐2015‐138, and S‐2018‐022 with DEW Scientific Research approval, Y26054‐6 and U26431‐1.

Whole blood samples for a subset of the koalas in this study were analyzed for KoRV (MLR *n* = 111 and KI *n* = 93), as reported in Fabijan et al. [4] and Stephenson et al. [8], and for *C. pecorum* using ocular and urogenital swabs (MLR 2016 *n* = 52, and KI *n* = 93), as reported in Fabijan et al. [9]. Haematologic reference intervals for a subset of the koalas in this study (KI and MLR 2016, *n* = 138) are reported in Fabijan et al. [17].

General serum biochemistry analyses were performed in March 2022 at Vetnostics laboratory, New South Wales, Australia, using a Cobas 8000 analyzer (Roche Diagnostics, Basel, Switzerland), obtaining data for the following analytes: albumin globulin ratio (A:G), albumin, alkaline phosphatase (ALP), alanine aminotransferase (ALT), aspartate aminotransferase (AST), bicarbonate, calcium, chloride, cholesterol, creatine kinase (CK), creatinine, gamma glutamyl transferase (GGT), globulins, glucose, magnesium, inorganic phosphate, symmetric dimethylarginine (SDMA), sodium, potassium, total protein (TP), triglycerides, and urea. Only kits provided by the manufacturer were used following the manufacturer's instructions. With the exception of SDMA, all reagents were from Roche (Roche Diagnostics, Basel, Switzerland). SDMA reagent was from Eurolyser (Eurolyser Diagnostica GmbH, Salzburg, Austria). Methodology and analyte performance are detailed in Table S1. Internal laboratory QC was performed for all analytes daily. Vetnostics is NATA‐accredited and is an active participant in the Royal College of Pathologists of Australasia Quality Assurance Programs (RCPAQAP) and VLA Veterinary Laboratory Association Quality Assurance Program (VLAQAP). Specific validation of these analytes for koalas has not been previously performed, but Vetnostics routinely analyses koala samples with clinically appropriate results achieved, including in normal animals.

Outlier values were determined and excluded using Reference Value Advisor (National Veterinary School of Toulouse, Toulouse, Haute‐Garonne, France) [22], Version 2.1, using Dixon's or Tukey's range tests. A tendency to retain rather than remove outlier values was adopted based on clinical evaluation of all biochemistry data of the individual. The outlier values detected and excluded were elevated CK in 10 koalas (2599, 2637, 2694, 2736, 2765, 3837, 4657, 9351, 17 711, 19 805 U/L), with four of these animals also showing high AST (values excluded: 88, 124, 130, 155 U/L), suggestive of capture myopathy which is common in wild marsupials. One koala also showed a markedly elevated urea value (21 mmol/L), which was excluded, as this was suggestive of dehydration, or possibly renal disease, although creatinine and SDMA were not elevated.

Following outlier exclusion, reference intervals (RIs) were generated for each analyte using the Reference Value Advisor Software, with the Anderson‐Darling test used to determine normality with *p* > 0.05. Confidence intervals (CI) of 90% were established to determine the upper and lower limits of the RIs, calculated using Reference Value Advisor according to ASVCP recommendations. Non‐parametric statistical methods were applied for establishing RI for *n* ≥ 120, with parametric or nonparametric methods for 40 ≤ *n* < 120, and bootstrap methods applied for CI calculation for non‐Gaussian data distribution [13]. For 20 ≤ *n* < 40, parametric or robust methods were applied to either Box‐Cox transformed or untransformed data. For 10 ≤ *n* < 20, descriptive statistics only were provided (mean, SD, median, min, max). These were based on guidelines developed by the International Federation for Clinical Chemistry and the Clinical and Laboratory Standards Institute [13].

To determine if pre‐analytical koala factors had an effect on biochemical values, two univariate general linear models were used with a type I sums of squares (*α* = 0.05) to compare the effects of both subclinical *C. pecorum* and KoRV infection with IBM SPSS v28 Statistical software (SPSS Inc., Chicago, IL, USA). In each model, pre‐analytical factors were ordered (1) population (MLR and KI) representing both geographic regions and different anesthetic protocols, (2) sex nested within population (as all KI koalas were female), (3) age group (juvenile—TWC I; young adult—TWC II, III, and IV; adult—TWC > IV), and (4) infectious agent detection, *C. pecorum* or KoRV (present or absent). All analytes and residuals were normally distributed. Partitioned reference intervals were then calculated for analytes with statistical significance based on these four factors.

## 3 Results

For this study, 206 serum samples were analyzed from free‐ranging, clinically healthy koalas, 113 from MLR (72 females and 41 males), and 93 koalas from KI (all females). Most of the koalas in the cohort were adults and young adults. In MLR, there were 40 adults, 56 young adults, 13 juveniles, and 4 of unrecorded age. In KI, 63 adults, 25 young adults, 4 juveniles and 1 of unrecorded age. Mean BCS for the different cohorts were 2.77 out of 3 in MLR and 2.92 in KI. Results of individual koalas can be found in Table S2.

Biochemical RIs generated for all study animals are presented in Table 1. Biochemical RIs for koalas that tested negative for both KoRV and *Chlamydia pecorum* (*n* = 69, 45 adults, 21 young adults, 2 juveniles, 1 unknown age; 64 females, 5 males) are also shown in Table 1. Histograms in Figures S1 and S2, respectively, show the distribution for each biochemical analyte from Reference Value Advisor for all study animals and those that tested negative. Biochemical values with statistical significance based on koala age are shown in Table 2, with histograms for juvenile koalas in Figure S3, and those with statistical significance based on KoRV and *Chlamydia* subclinical infection status (positive or negative), sex, and population (geographic location and anesthetic differences), are shown in Table S3.

**TABLE 1:** Serum biochemistry reference intervals (RIs) of all koalas and those that tested negative for koala retrovirus (KoRV) and *Chlamydia pecorum* .

Analyte, units	Cohort	*n* ^a^	Mean	SD	Median	Min	Max	Anderson–Darling test	Dist.^b^	Method^c^	LRL of RI	URL of RI	90% CI of LRL	90% CI of URL
Sodium, mmol/L	All	195	141	4	141	131	153	0.019	NG	NP	133	150	133–134	148–151
	Neg^d^	66	139.5	3.7	139	131	149	0.484	G	P	132	147	131–133	146–148
Potassium, mmol/L	All	195	5.0	0.8	4.9	3.6	8.9	0.000	NG	NP	3.8	7.2	3.6–4.0	6.4–7.7
	Neg	66	5.2	0.9	5.1	3.6	8.9	0.003	NG	NP	3.8	7.8	3.6–4.2	6.4–8.9
Chloride, mmol/L	All	195	101	4	100	89	113	0.023	NG	NP	94	108	91–95	107–110
	Neg	66	99.5	3.5	100	91	109	0.123	G	P	93	107	91–94	105–108
Bicarbonate, mmol/L	All	196	19	3	19	12	27	0.010	NG	NP	14	25	12–14	25–26
	Neg	67	18	3	18	12	25	0.012	NG	NP	13	25	12–14	23–25
Magnesium, mmol/L	All	196	1.05	0.22	1.05	0.52	1.63	0.944	G	NP	0.64	1.48	0.56–0.67	1.42–1.56
	Neg	66	1.01	0.26	0.97	0.52	1.63	0.177	G	P	0.49	1.53	0.40–0.58	1.45–1.62
Calcium, mmol/L	All	195	2.77	0.23	2.76	2.24	4.19	0.000	NG	NP	2.37	3.13	2.26–2.45	3.05–4.1
	Neg	66	2.83	0.14	2.83	2.26	3.08	0.033	NG	NP	2.39	3.06	2.26–2.64	3.02–3.08
Phosphate, mmol/L	All	194	1.33	0.4	1.28	0.29	2.75	0.002	NG	NP	0.63	2.47	0.49–0.75	2.05–2.55
	Neg	65	1.43	0.38	1.43	0.49	2.55	0.089	G	P	0.67	2.20	0.55–0.80	2.06–2.33
Urea, mmol/L	All	205	3.1	1.6	2.9	0.2	9.2	0.001	NG	NP	0.6	6.5	0.3–0.9	6–8.3
	Neg	69	2.8	1.6	2.6	0.2	8.3	0.031	NG	NP	0.5	6.6	0.2–0.8	5.7–8.3
Creatinine, μmol/L	All	206	95	17	94	54	151	0.018	NG	NP	63	131	55–71	126–147
	Neg	69	94	13	95	54	130	0.097	G	P	67	121	62–71	116–125
SDMA, μg/dL	All	193	16.7	4.9	16	9	31	0.000	NG	NP	9.9	28	9–10	26–29
	Neg	64	19.4	4.8	18	11	31	0.007	NG	NP	11.6	29.1	11–12.9	28–31
AST, U/L	All	196	24	12	20	9	68	0.000	NG	NP	10	57	10–11	52–64
	Neg	65	26	12	23	9	63	0.000	NG	NP	11	57	9–13	51–63
CK, U/L	All	187	505	429	339	99	2148	0.000	NG	NP	126	1956	99–135	1526–2148
	Neg	62	671	465	508	167	1985	0.000	NG	NP	170	1967	167–187	1562–1985
GGT, U/L	All	197	12	3	12	6	19	0.000	NG	NP	7	17	6–7	16–19
	Neg	67	13	3	14	7	19	0.019	NG	NP	7	19	7–9	17–19
ALP, U/L	All	199	175	141	119	17	625	0.000	NG	NP	30	576	19–40	481–598
	Neg	68	206	140	146	30	576	0.000	NG	NP	46	548	30–57	467–576
ALT, U/L	All	200	10	7	9	3	63	0.000	NG	NP	5	24	3–5	22–49
	Neg	68	13	8	11	5	63	0.000	NG	NP	5	36	5–6	22–63
Glucose, mmol/L	All	194	5.7	1.5	5.5	2.3	10.7	0.039	NG	NP	2.6	8.6	2.3–3.5	8.2–10.7
	Neg	65	6.0	1.4	5.7	3.6	10.7	0.208	G	P	3.1	8.8	2.7–3.6	8.3–9.3
Triglycerides, mmol/L	All	195	1.2	0.5	1.2	0.3	2.7	0.001	NG	NP	0.4	2.4	0.4–0.5	2.3–2.6
	Neg	65	1.1	0.4	1.0	0.3	2.2	0.004	NG	NP	0.4	2.1	0.3–0.6	1.9–2.2
Cholesterol, mmol/L	All	196	2.0	0.5	1.9	1.1	3.7	0.000	NG	NP	1.2	3.1	1.2–1.4	2.8–3.5
	Neg	66	2.1	0.5	2.1	1.1	3.4	0.362	G	P	1.2	3.0	1.1–1.4	2.9–3.2
Total protein, g/L	All	205	61	6	61	46	75	0.244	G	NP	48	73	46–51	70–75
	Neg	68	64	6	65	49	75	0.062	G	P	52	77	49–54	75–79
Albumin, g/L	All	203	40	4	40	26	47	0.001	NG	NP	32	46	28–33	45–46
	Neg	68	41	3	41	31	47	0.076	G	P	35	47	34–36	46–48
Globulin^e^, g/L	All	203	21	4	21	13	33	0.025	NG	NP	14	30	13–15	29–32
	Neg	68	23	4	23	13	33	0.699	G	P	13	32	13–16	30–33
A:G^e^	All	203	1.9	0.3	1.9	1.1	3	0.143	G	NP	1.2	2.7	1.2–1.4	2.5–2.9
	Neg	68	1.9	0.4	1.8	1.2	2.9	0.149	G	P	1.2	2.6	1.0–1.3	2.4–2.7

^a^ Number of koala samples analyzed for each analyte varies due to either sample volume available or outlier exclusion.

^b^ Distribution. G = Gaussian, NG = Non‐Gaussian.

^c^ Statistical method for establishing RI: P, parametric; NP, nonparametric; R, robust; T, transformed.

^d^ Neg = koalas that tested negative to both koala retrovirus (KoRV) and *Chlamydia pecorum* by PCR.

^e^ Calculated analytes (others measured). Globulin = total protein‐albumin. A:G = albumin/globulin.

**TABLE 2:** Koala serum biochemistry variables based on age groups of koalas for analytes with statistical significance.

Analyte^a^	Units	Age^b^	*N* ^c^	Mean	SD	Median	Min	Max	Anderson–Darling test	Dist.^d^	Method^e^	LRL of RI	URL of RI	90% CI of LRL	90% CI of URL	*p* ^f^
Potassium	mmol/L	J	14	4.94	0.45	4.9	4	5.6								
		YA	78	5.1	0.96	4.9	3.6	8.9	0.000	NG	NP	3.8	7.73	3.6–4.0	6.86–8.9	0.019
		A	99	4.93	0.72	4.9	3.6	7.3	0.002	NG	NP	3.7	6.9	3.6–4.0	6.1–7.3	
Chloride	mmol/L	J	14	99	3	99	92	104								
		YA	78	101	4	101	89	110	0.265	G	NP	94	108	89–95	107–110	0.027
		A	99	101	4	100	91	113	0.096	G	NP	94	109	91–95	108–113	
Magnesium	mmol/L	J	15	1.11	0.2	1.04	0.89	1.56								
		YA	78	1.11	0.19	1.08	0.7	1.63	0.294	G	NP	0.73	1.48	0.7–0.83	1.43–1.63	0.003
		A	99	0.98	0.23	0.96	0.52	1.53	0.310	G	NP	0.57	1.46	0.52–0.64	1.37–1.53	
**Phosphate**	mmol/L	J	14	1.79	0.42	1.71	1.13	2.75								
		YA	78	1.31	0.43	1.26	0.29	2.55	0.011	NG	NP	0.60	2.46	0.29–0.79	2.05–2.55	< 0.001
		A	98	1.28	0.32	1.26	0.49	2.51	0.325	G	NP	0.66	2.02	0.49–0.75	1.74–2.51	
Creatinine	μmol/L	J	17	81	15	79	57	111								
		YA	81	97	16	96	54	151	0.038	NG	NP	71	146	54–75	122–151	< 0.001
		A	103	96	16	95	55	134	0.108	G	NP	61	131	55–74	126–134	
CK	U/L	J	15	684	335	557	300	1460								
		YA	77	449	407	286	99	1960	0.000	NG	NP	123	1954	99–135	1227–1960	0.006
		A	91	535	458	359	110	2148	0.000	NG	NP	119	2070	110–144	1470–2148	
GGT	U/L	J	15	11	2	11	8	15								
		YA	79	12	3	12	7	18	0.028	NG	NP	7	17	7–7	16–18	0.049
		A	99	13	3	13	6	19	0.004	NG	NP	6	18	6–8	17–19	
**ALP**	U/L	J	15	412	143	364	74	625								
		YA	80	194	147	136	34	537	0.000	NG	NP	40	511	34–49	446–537	< 0.001
		A	100	126	103	92	17	598	0.000	NG	NP	24	510	17–35	335–598	
**TP**	g/L	J	16	52	4	54	46	60								
		YA	81	60	5	60	46	74	0.701	G	NP	48	70	46–52	67–74	< 0.001
		A	103	64	6	65	49	75	0.045	NG	NP	51	74	49–54	72–75	
**Globulin** ^g^	g/L	J	16	16	2	15	13	19								
		YA	81	20	3	20	13	30	0.056	G	NP	14	27	13–16	26–30	< 0.001
		A	102	24	4	23	16	33	0.039	NG	NP	17	31	16–18	30–33	
**A:G** ^g^		J	16	2.4	0.3	2.4	2.0	3								
		YA	81	2.0	0.3	2.0	1.2	2.9	0.420	G	NP	1.5	2.7	1.2–1.5	2.4–2.9	< 0.001
		A	102	1.8	0.3	1.8	1.1	2.4	0.756	G	NP	1.2	2.3	1.1–1.3	2.2–2.4	

^a^ Analytes in bold indicate those for which partitioned values should be used in a clinical setting.

^b^ Juvenile koala RI histograms from Reference Value Advisor software are shown in Figure S2.

^c^ Number of koala samples analyzed for each analyte varies due to either sample volume available or outlier exclusion.

^d^ Distribution. G = Gaussian, NG = Non‐Gaussian.

^e^ Statistical method for establishing RI: P, parametric; NP, nonparametric; R, robust; T, transformed.

^f^ *p‐*value result for overall analysis between the three groups, see text for pairwise analyses between groups.

^g^ Calculated analytes (others measured). Globulin = total protein‐albumin. A:G = albumin/globulin.

In Table 2, of clinical, as well as statistical significance, juvenile animals were found to have significantly higher values of ALP than young adults (*p* < 0.001) and adults (*p* < 0.001), phosphate higher than young adults (*p* < 0.001) and adults (*p* < 0.001) and A:G higher than young adults (*p* < 0.001) and adults (*p* < 0.001). Adults had higher values of total protein (*p* < 0.001) and globulins (*p* < 0.001). Partitioned RIs should be used for these analytes in a clinical setting.

For other statistically significant analyte differences with less diagnostic importance in a clinical setting, juveniles had CK values slightly higher than young adults (*p* = 0.015) and adults (*p* = 0.002). Chloride was marginally lower in juveniles than both young adults (*p* = 0.043) and adult animals (*p* = 0.008), while young adults and adults had no significant differences from each other. Juveniles had slightly lower creatinine than both young adults and adults (*p* < 0.001). Adult individuals had marginally higher GGT than juveniles (*p* = 0.048) and young adults (*p* = 0.043), while juveniles and young adults had no differences. Adults had marginally lower potassium (*p* = 0.005) and magnesium (*p* < 0.001) than young adults (Table 2).

In Table S3, for subclinical infection with KoRV, glucose was found to be marginally higher in positive koalas. The results for subclinical infection with *C. pecorum* showed that negative individuals had marginally higher GGT compared to positive individuals (Table S3). All other analytes were not statistically significant. Based on the sex of koalas, MLR females had marginally higher albumin and GGT, while MLR male koalas had higher ALP, bicarbonate, magnesium, and triglycerides.

Based on population, timing, and method of anesthesia, KI koalas anesthetized through mask delivery of isoflurane several hours after capture presented statistically higher albumin, ALP, ALT, AST, calcium, cholesterol, CK, GGT, globulins, glucose, phosphate, potassium, SDMA, TP, and urea compared with MLR koalas anesthetized soon after capture with injectable agents, in which bicarbonate, chloride, magnesium, sodium, and triglycerides were higher. These results may assist in the interpretation of results in future research studies (Table S3).

## 4 Discussion

This study presented updated biochemical RIs of koalas, based on data from 206 free‐ranging koalas from SA. The koalas in this study were clinically healthy and in good or excellent body condition, and of known subclinical KoRV and *C. pecorum* status.

The RI's developed in this study for all koalas are similar to those previously published by Canfield et al. [18], based on 45 koalas from the northern population. Several of the analytes in the current study showed either slightly narrower reference intervals (bicarbonate, urea), lower upper reference limits of the RI (creatinine, AST, ALT, triglycerides, total protein, albumin, globulin) or higher upper reference limits (calcium, ALP). Also, additional analytes were included in the current study (magnesium, SDMA, CK, glucose). ALT (alanine aminotransferase) and AST (aspartate aminotransferase) were the analytes that showed the most marked differences, showing much lower upper reference limits of the RIs in the current study [18]. It is possible that these differences have a basis in a more detailed health assessment of the koalas in the current study since some of the animals' health statuses were not reported in the previous study, and *C. pecorum* and KoRV were not tested. Differences may also be attributed to the fact that this study sampled a larger number of animals (*n* = 206) than the previous study (*n* = 45), and that interlaboratory variability exists, with advancement of some assays over time leading to different analytical methods being used. However, it is not possible to exclude that differences are due to the large sampling to analysis interval in the current study, since samples were stored for a long duration.

Although only clinically healthy animals were included in this study, subclinical infections of *C. pecorum* and KoRV are frequently found in healthy koalas [4, 9], and this was associated with no clinically significant differences in biochemical analytes. Also, the RIs for 69 koalas negative for both infections showed few differences from the RIs of the entire cohort. The prevalence of *C. pecorum* infection in the MLR population is high but is largely subclinical, with only a low number of koalas showing clinical chlamydiosis. In northern koalas with overt clinical chlamydiosis, uncomplicated cystitis has not previously been reported to cause any changes in biochemical analytes; however, complicated cystitis can lead to azotaemia [23]. In the current study, in those animals free from *C. pecorum* infection, only marginally higher levels of GGT were statistically significant compared with positive animals; therefore, this minor difference was not considered to be clinically significant. KoRV is ubiquitous in northern populations, and although it is also high in southern populations, most of the koalas in SA have lower proviral loads [4], and there is fragmented expression and possible dysfunction of KoRV in MLR koalas [21]. In this study, KoRV‐positive koalas had marginally higher glucose levels compared with KoRV‐negative koalas. Besides this minor finding, no other statistically significant differences were found in biochemical analytes between positive and negative animals. This suggests that KoRV is not affecting the health of SA koalas in the absence of KoRV‐related clinical diseases such as lymphosarcoma/leukemia. Unfortunately, the high prevalence of both of these infections makes it difficult to include only negative animals in any koala research study. However, as South Australian populations have a lower prevalence of infections, we were able to generate RIs for 69 koalas negative for both KoRV and *Chlamydia pecorum* to support that there was little impact of subclinical infection on koala health.

There were, however, several significant differences in serum biochemistry based on koala age. Some of the age‐related differences were statistically and clinically significant (Table 2). Clinically significant differences included that juvenile and young adult koalas had elevated A:G, ALP, and phosphate, and lower globulins and total protein, compared to adults. These findings are in line with other studies that report similar changes in koalas from the northern population [18], and other domestic and wildlife species [24, 25]. Both the Dickens [15] and Canfield et al. [18] studies reported high ALP values in juvenile koalas, which is related to increased bone metabolism in growing animals [20], and paralleled by elevated phosphate, also associated with bone growth [26]. Furthermore, the elevated level of total proteins in adults can be attributed to higher globulin concentration in adult animals, which in turn affects the A:G ratio. Juvenile koalas had significantly lower creatinine than both young adults and adults, which may relate to lower muscle mass in young animals, as is recognized in other species.

The comparison based upon sex of koalas was carried out between females and males of the MLR population to eliminate the bias created by population differences, as male koalas were not sampled in the KI population. Previous studies do not report differences between male and female koalas [18, 27], and whilst statistically significant differences were found (Table S3), most were not clinically important as they were only marginally different.

Some biochemical values showed differences between the MLR and KI populations (Table S3), which could be attributed to differences between populations, sampling intervals, or anesthesia methods. In this study, for the KI koalas, koalas were captured and housed for several hours before surgical sterilization using isoflurane gas anesthesia [4], whereas in MLR, koalas were captured and soon after sampled under anesthesia using injectable agents [4, 19]. Minor differences in CK and AST may reflect the longer period between capture and sampling in the KI koalas, allowing biochemically detectable increases in soft tissue enzymes to occur; furthermore, relative decreases in bicarbonate in this group may be associated with metabolic titrational lactic acidosis. Minor increases in albumin, urea, total protein, phosphate, and SDMA may also reflect variation in hydration status, associated with decreased feed or water intake in the time held prior to anesthesia in the KI group. Also, it is not possible to discriminate between the differences based on geographical locations (MLR and KI), sampling, or anesthesia, so further studies would be required to investigate these effects. Also, research on the effect of sample storage duration on biochemical analytes would be valuable, given that the sample‐analysis interval was lengthy in this study.

Another condition to consider is oxalate nephrosis, which has a high prevalence in koalas from the MLR population, with 32% of necropsied koalas showing consistent renal histopathologic changes [10]. Affected koalas have elevated SDMA concentration, and usually, azotemia is present [11, 12]. In the current study, no koalas showed evidence of renal dysfunction (except the one excluded koala, see Methods). However the SDMA RI was found to be slightly higher (9.9–28 μg/dL) compared with the previous RI developed in koalas (2.4–22.9 μg/dL); yet this was calculated from samples of only 22 necropsied individuals [12], with a different assay, and SDMA dispersion can be marked in other species [28]. This higher RI could also be related to subclinical oxalate nephrosis in some koalas, with urine samples unable to be collected from any koalas to check for crystals [11], mainly due to urination at, or following, capture (author obs.). However the difference is relatively minor given that affected koalas have been found with a mean SDMA of 38.7 μg/dL (*n* = 19) [12].

In conclusion, this study presents the most comprehensive set of serum biochemical reference intervals for a large cohort of clinically healthy, free‐ranging koalas from the SA region. Changes in blood biochemistry related to the age of koalas due to growth, as in other species, were the main significant differences found, with other factors such as sex and KoRV/ *C. pecorum* infection status having minimal or no effect from a clinical perspective. The findings of this study will be valuable for health monitoring and disease research, both for individual koalas and for populations across Australia.

## Conflicts of Interest

The authors declare no conflicts of interest.

## Supporting information


**Table S1.** Methodology and analyte performance.

**Table S2.** Raw data. Yellow indicates removed outliers and blank cells indicate insufficient sample volume for analysis.

**Figure S1.** Histograms of analyte distribution for all koalas from Reference Value Advisor software.

**Figure S2.** Histograms of analyte distribution for koalas negative for koala retrovirus (KoRV) and *Chlamydia pecorum* from Reference Value Advisor software.

**Figure S3.** Histograms of analyte distribution of juvenile koalas from Reference Value Advisor software.

**Table S3.** Partitioned biochemical variables on the basis of sex, infection status, and location for those analytes with statistical significance.
