# Supplementary material for: Biochemical Reference Intervals of Free‐Ranging Koalas ( Phascolarctos cinereus ) in South Australia
Source: Vet Clin Pathol. 2025 Jul 2;54(3):300–8. doi: 10.1111/vcp.70024 (PMC12444011; doi:10.1111/vcp.70024)
Supplement: Supplementary file 1 — Table S1. Methodology and analyte performance. [file VCP-54-300-s004.docx]

| **Analyte** | **Units** | **Method** | **MU %** | **SD** | **Mean** |
| --- | --- | --- | --- | --- | --- |
| **Albumin** | g/L | Bromocresol green binding | 3.4 | 0.8 | 44.3 |
| **ALP** | IU | p-NPP hydrolyzation | 7.7 | 14.6 | 380 |
| **ALT** | IU | Catalyzation | 3.4 | 1.4 | 84.2 |
| **AST** | IU | Catalyzation | 3.3 | 3.2 | 197 |
| **Bicarbonate** | mmol/L | Enzymatic (colorimetric) | 7.2 | 1.3 | 35.4 |
| **Calcium** | mmol/L | Colorimetric (CPC) | 1.8 | 0.03 | 3.18 |
| **Chloride** | mmol/L | Ion selective electrode | 1.5 | 0.8 | 104 |
| **Cholesterol** | mmol/L | Enzymatic (colorimetric) | 4.2 | 0.1 | 6.8 |
| **CK** | IU | Enzymatic (colorimetric) | 2.5 | 6.1 | 486.8 |
| **Creatinine** | umol/L | Jaffe reaction | 6.8 | 16.4 | 480.3 |
| **GGT** | IU | Enzymatic (colorimetric) | 3.9 | 3.6 | 182.8 |
| **Glucose** | mmol/L | Hexokinase | 3.4 | 0.3 | 15.5 |
| **Magnesium** | mmol/L | Colorimetric | 3.2 | 0.03 | 1.6 |
| **Phosphate** | mmol/L | Phosphomolybdate | 3.0 | 0.04 | 2.45 |
| **Potassium** | mmol/L | Ion selective electrode | 1.1 | 0.0 | 6.8 |
| **Protein** | g/L | Biuret | 3.1 | 1.1 | 69.6 L |
| **SDMA** | ug/dL | Immunoturbimetric | 15.8 | 4.1 | 51.4 |
| **Sodium** | mmol/L | Ion selective electrode | 1.0 | 0.8 | 159.0 |
| **Triglycerides** | mmol/L | LPL dehydrolyzation | 2.6 | 0.03 | 2.13 |
| **Urea** | mmol/L | Coupled-enzyme reaction | 5.8 | 0.5 | 15.5 |
|  |  |  |  |  |  |

**Table S1.** Methodology and analyte performance.
