# Supplementary figures and images for: Biochemical Reference Intervals of Free‐Ranging Koalas ( Phascolarctos cinereus ) in South Australia
Source: Vet Clin Pathol. 2025 Jul 2;54(3):300–8. doi: 10.1111/vcp.70024 (PMC12444011; doi:10.1111/vcp.70024)

FIGURE S1. Histograms of analyte distribution for all koalas from RefVal software.


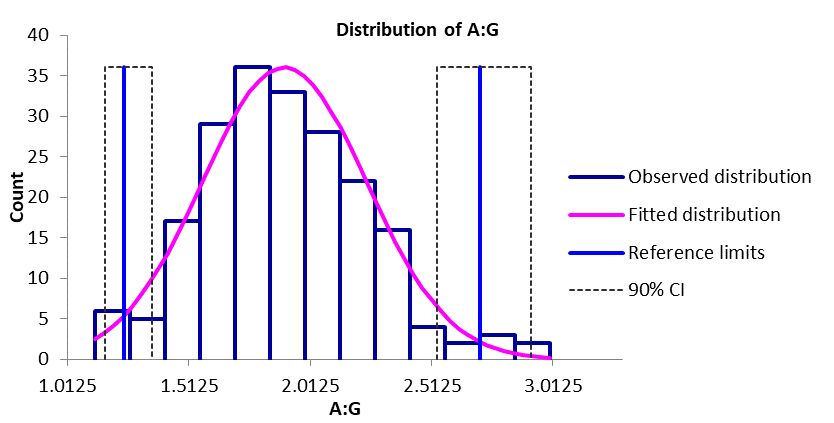

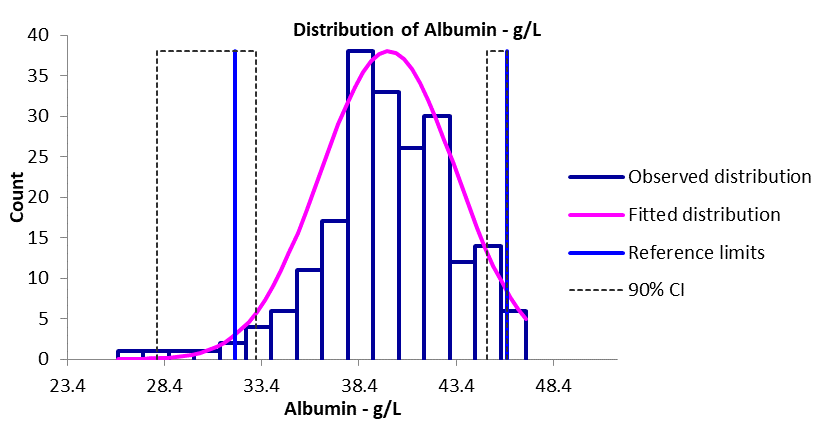


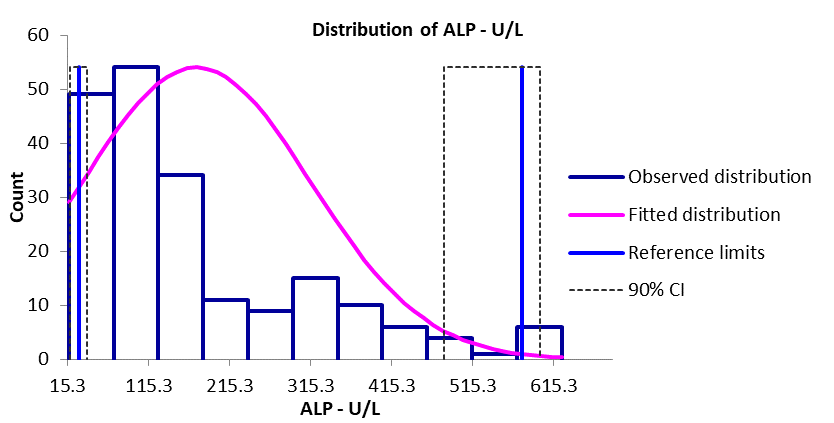

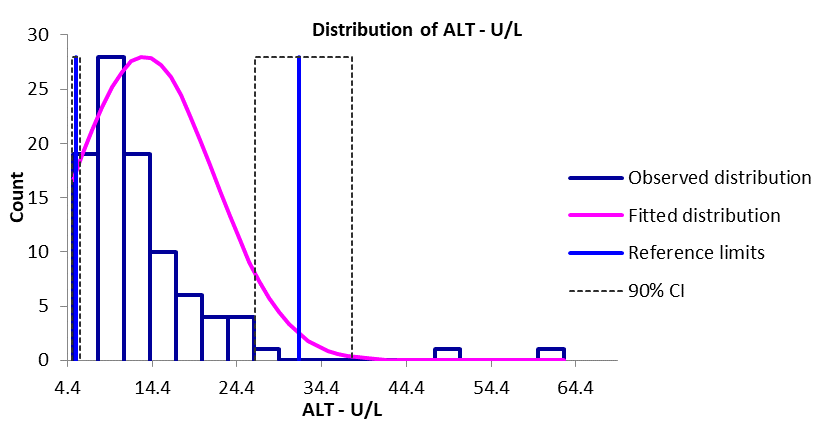


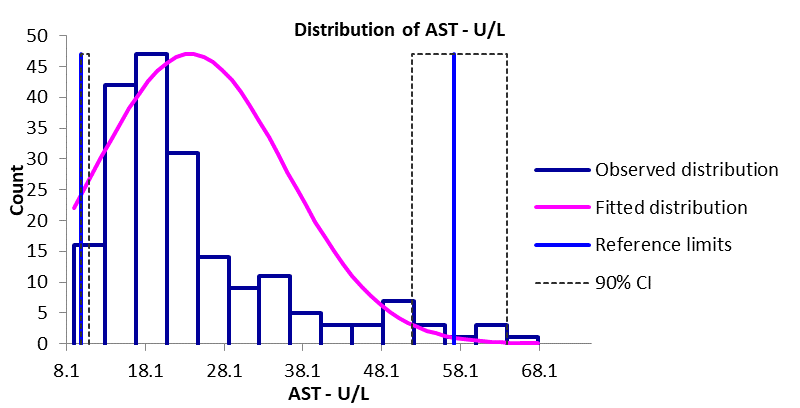

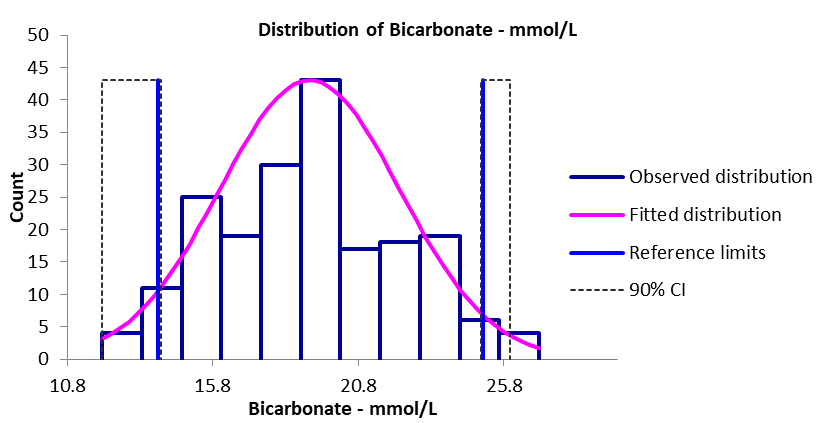


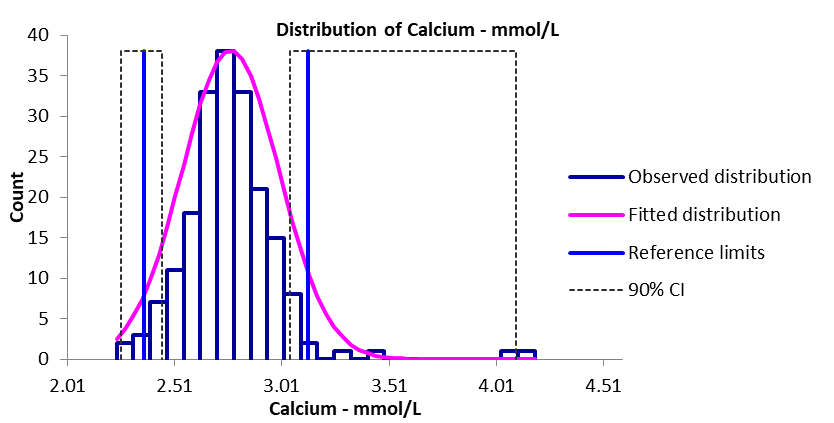

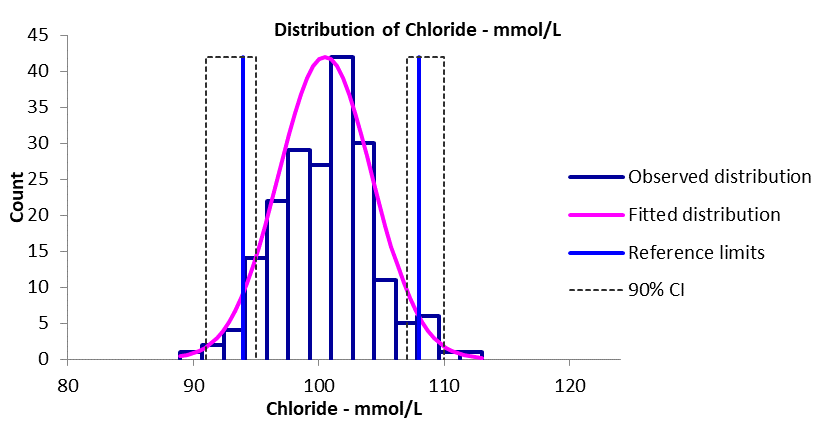


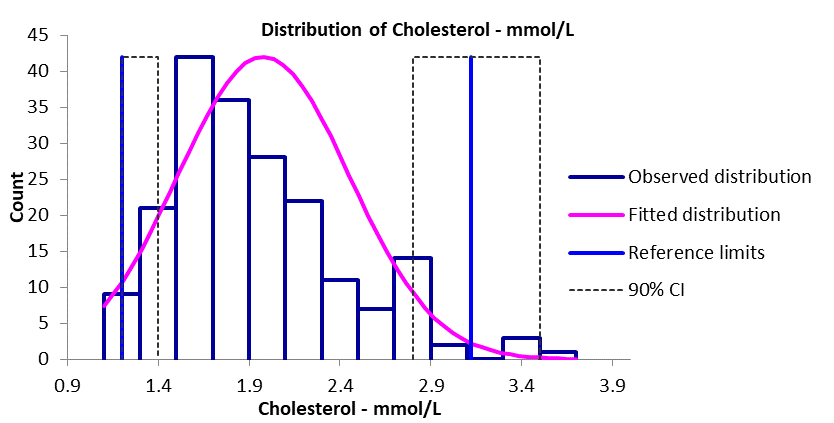

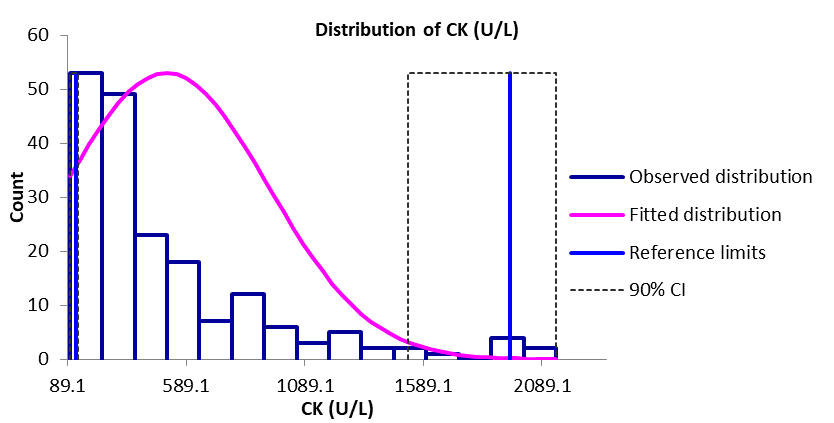


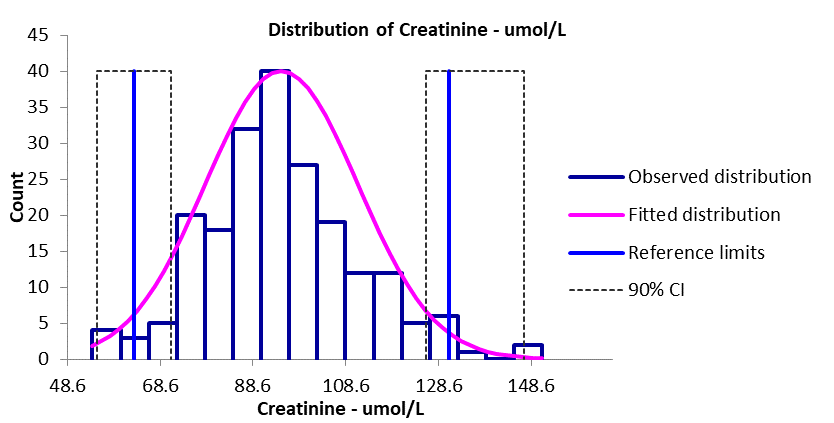

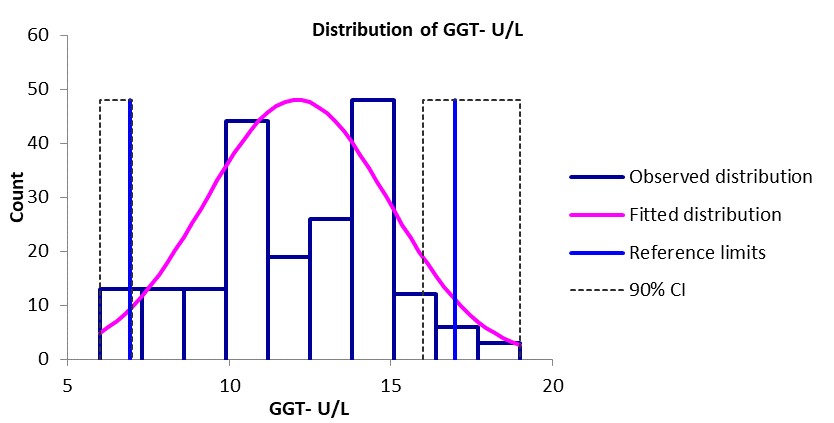


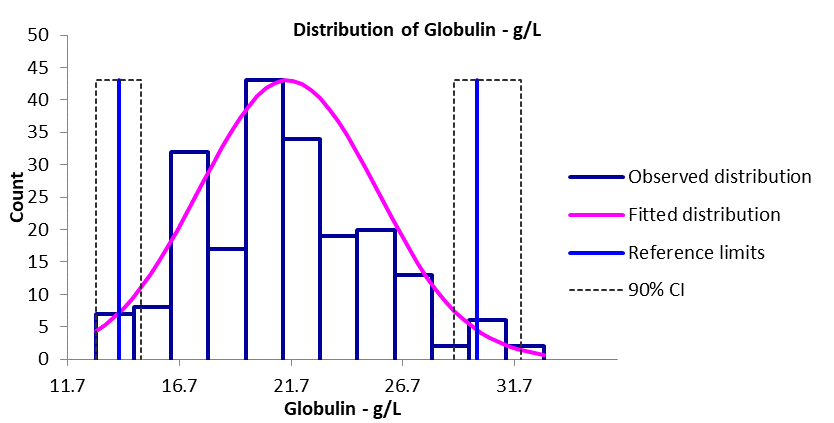

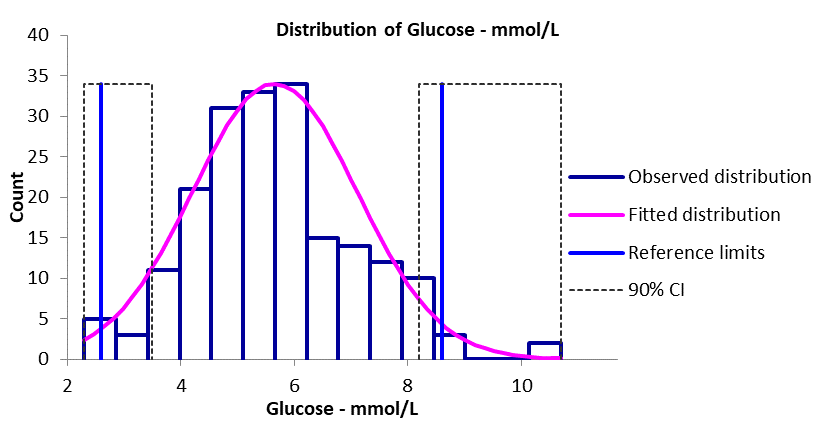


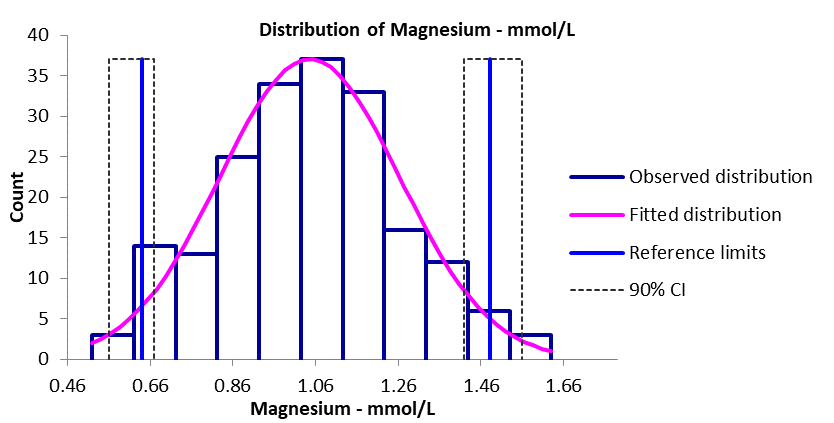

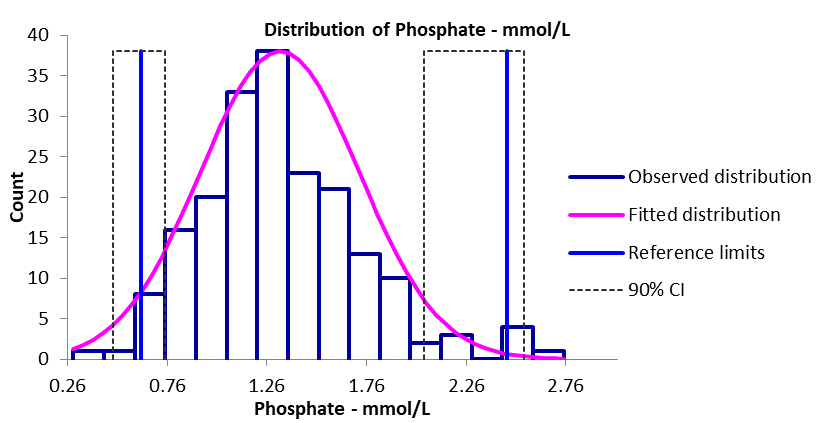


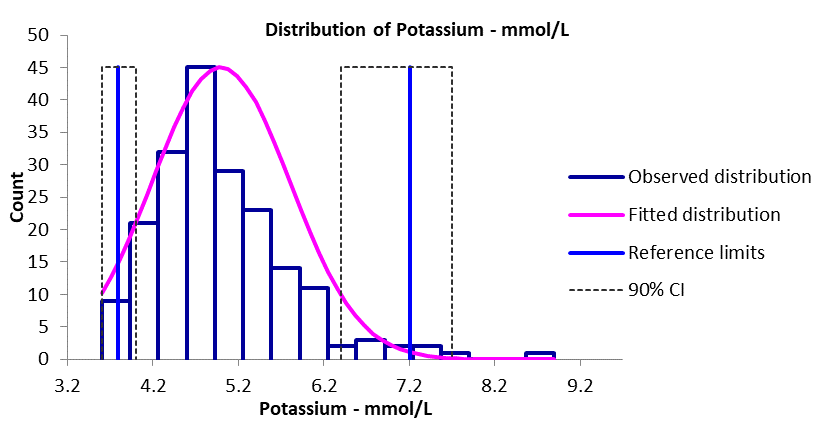

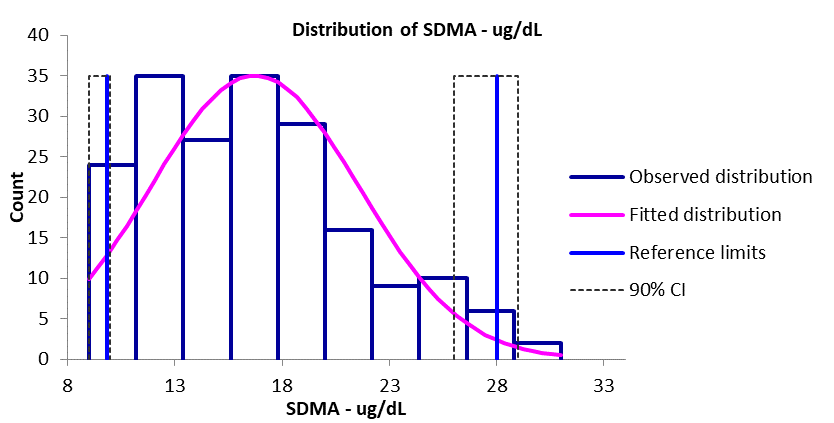


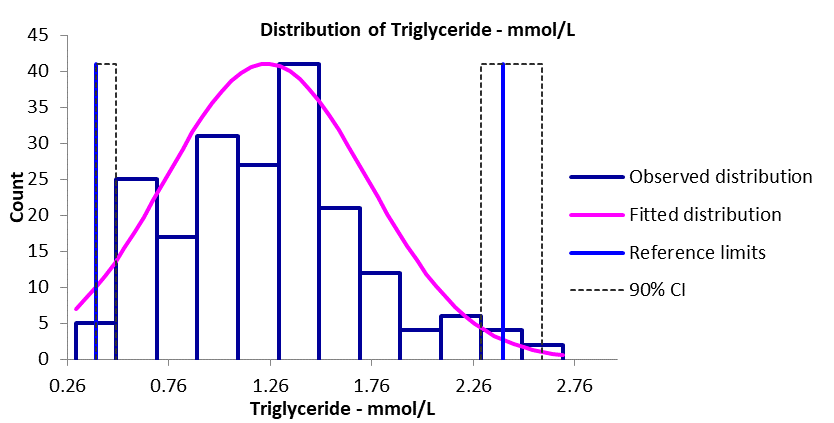

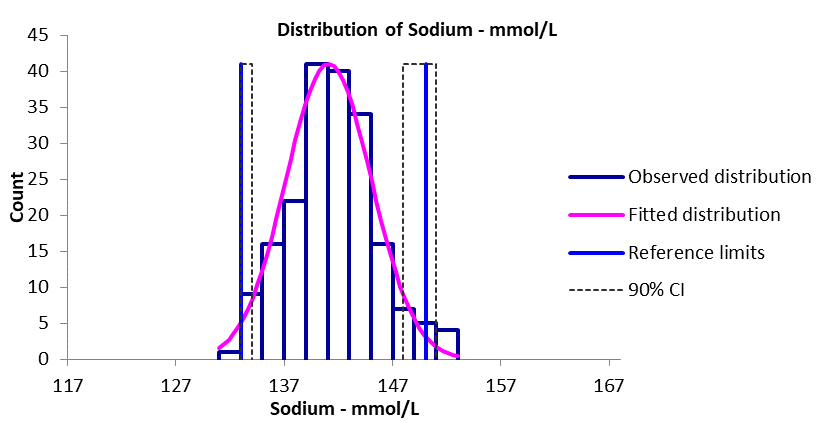


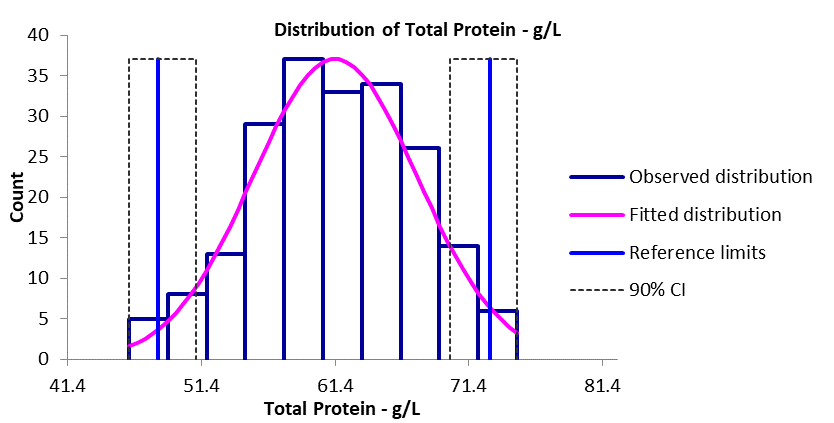

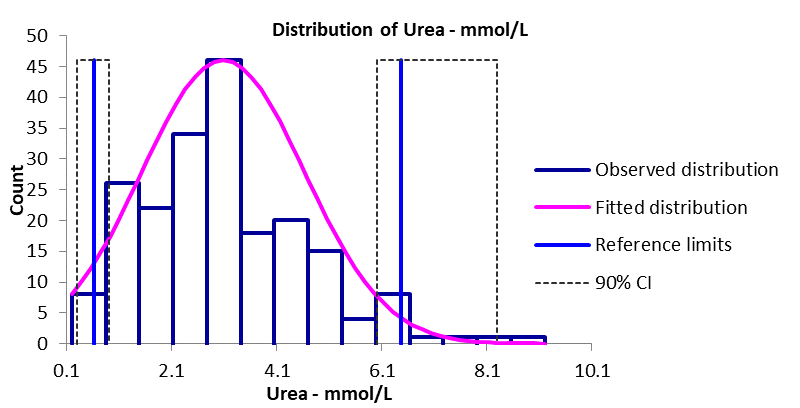

Supplement: Supplementary file 3 — Figure S1. Histograms of analyte distribution for all koalas from Reference Value Advisor software. [file VCP-54-300-s002.docx]
