# Supplementary material for: Biochemical Reference Intervals of Free‐Ranging Koalas ( Phascolarctos cinereus ) in South Australia
Source: Vet Clin Pathol. 2025 Jul 2;54(3):300–8. doi: 10.1111/vcp.70024 (PMC12444011; doi:10.1111/vcp.70024)

FIGURE S2. Histograms of analyte distribution for koalas negative for both KoRV and *Chlamydia pecorum*.


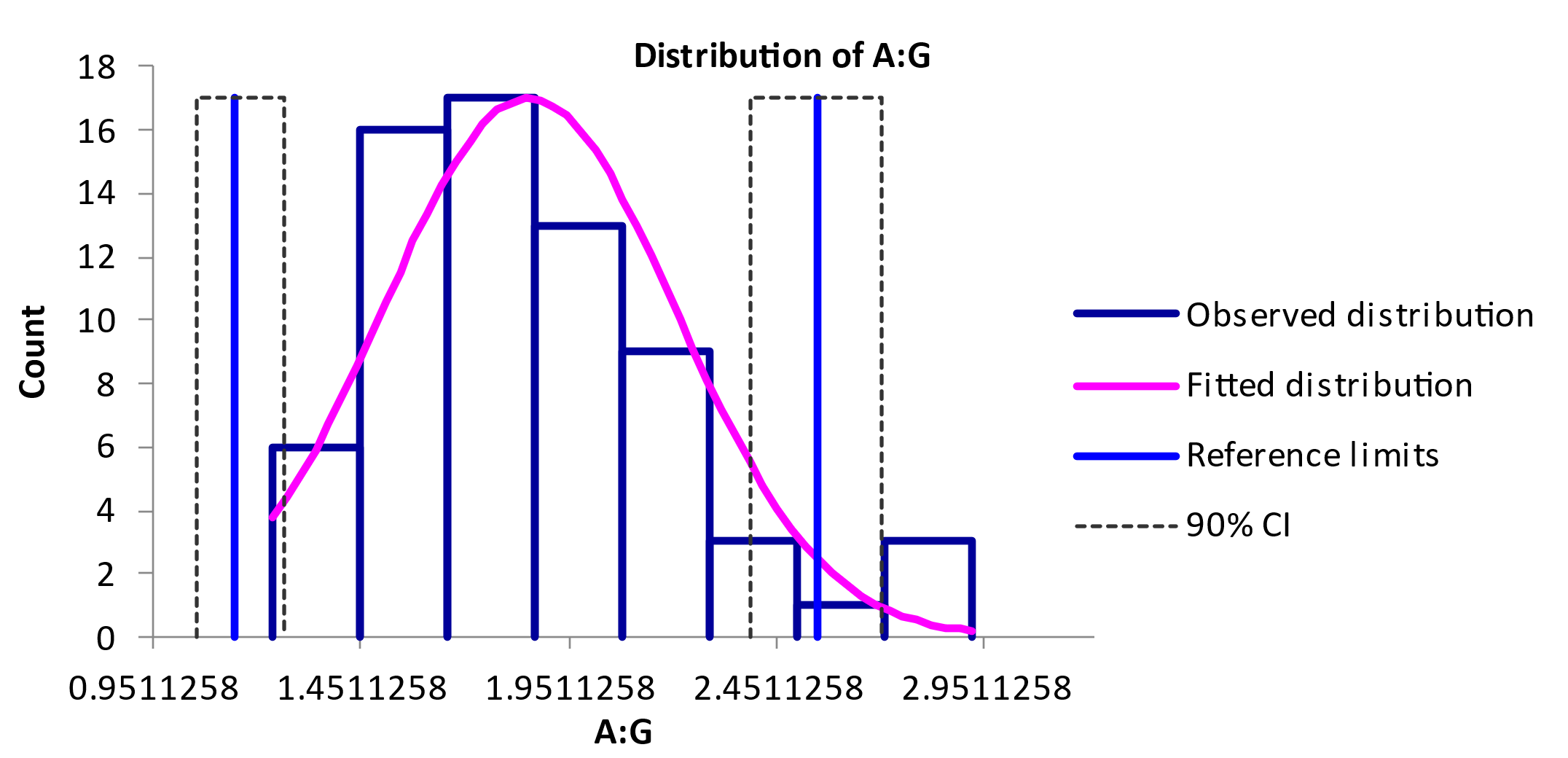

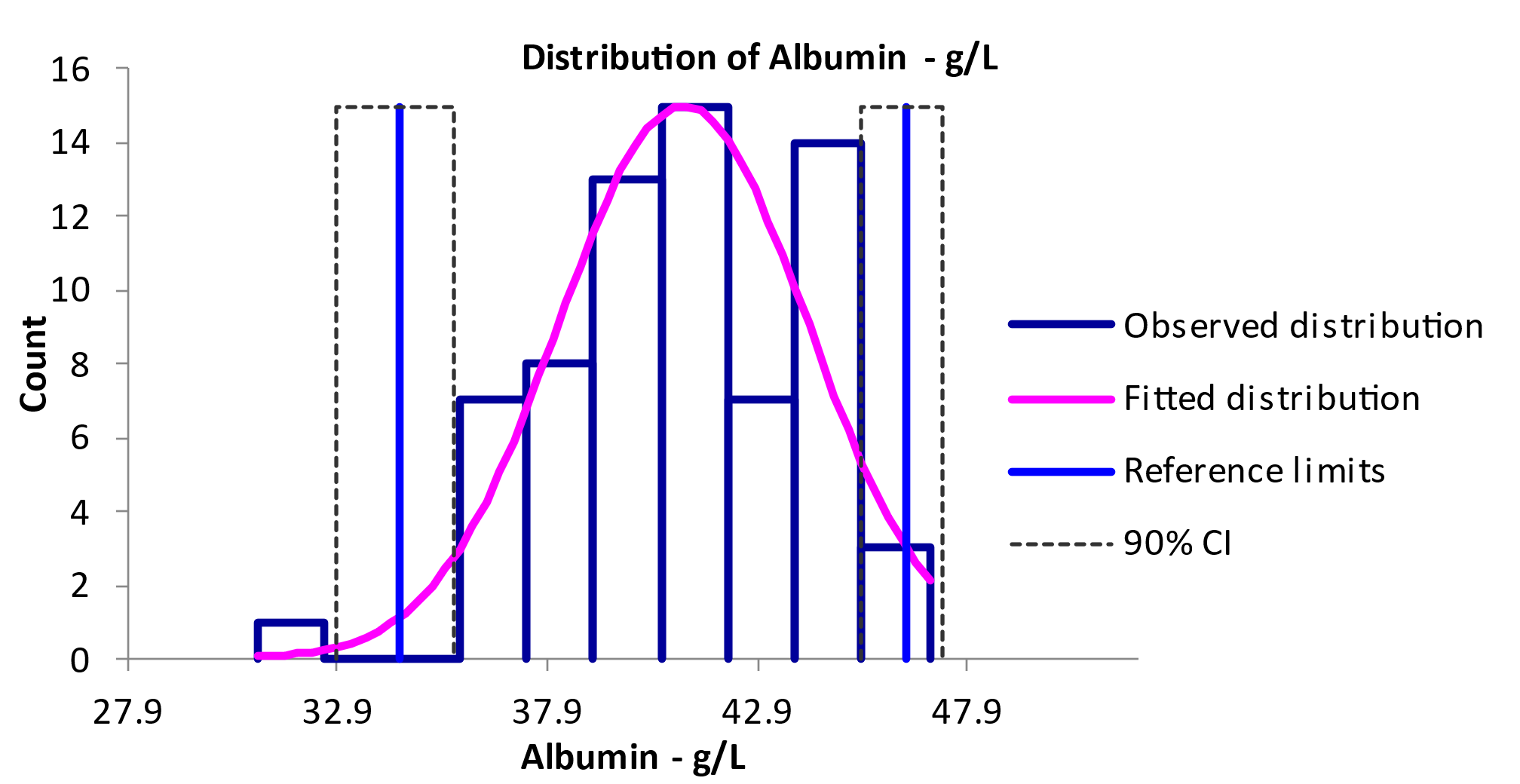


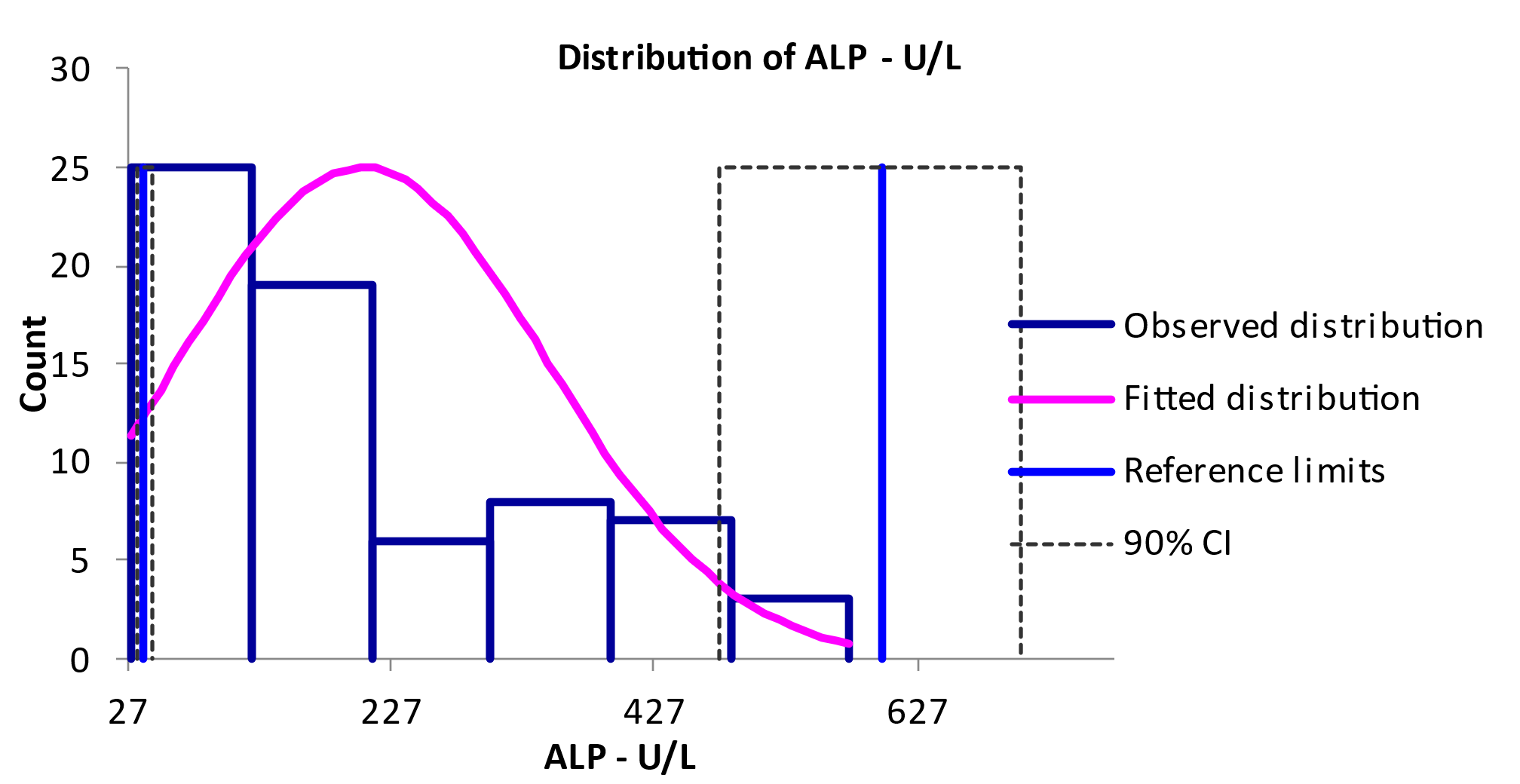

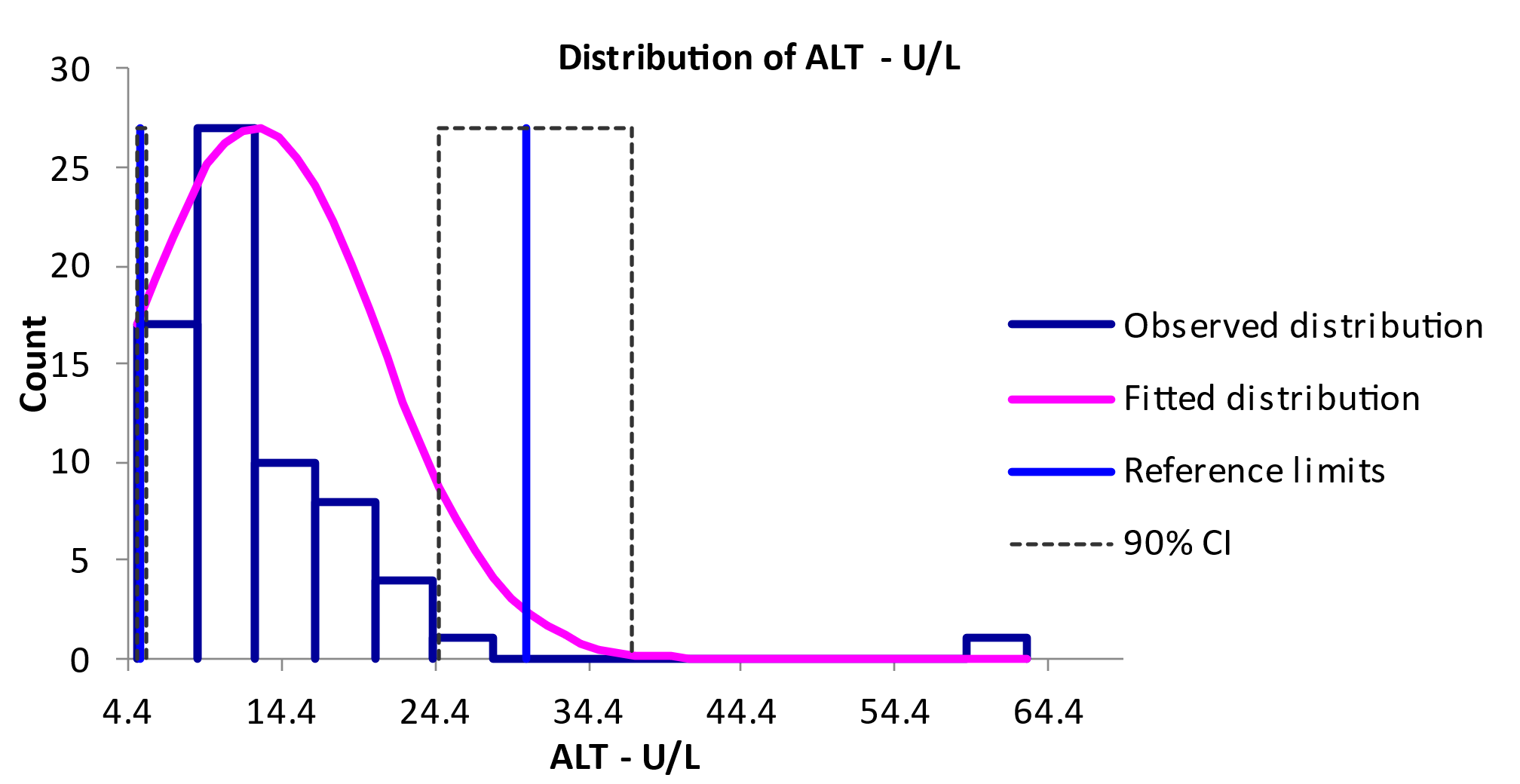


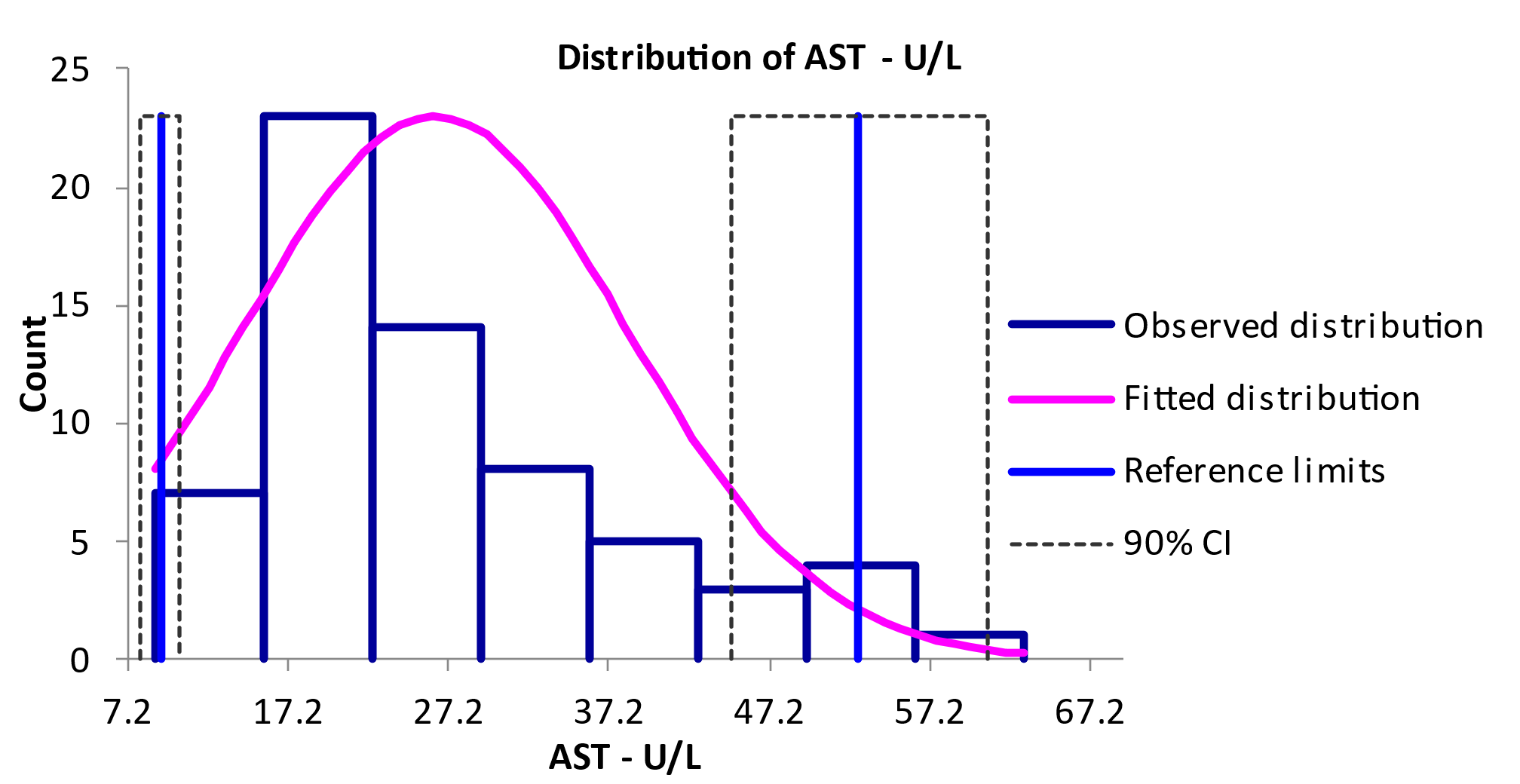


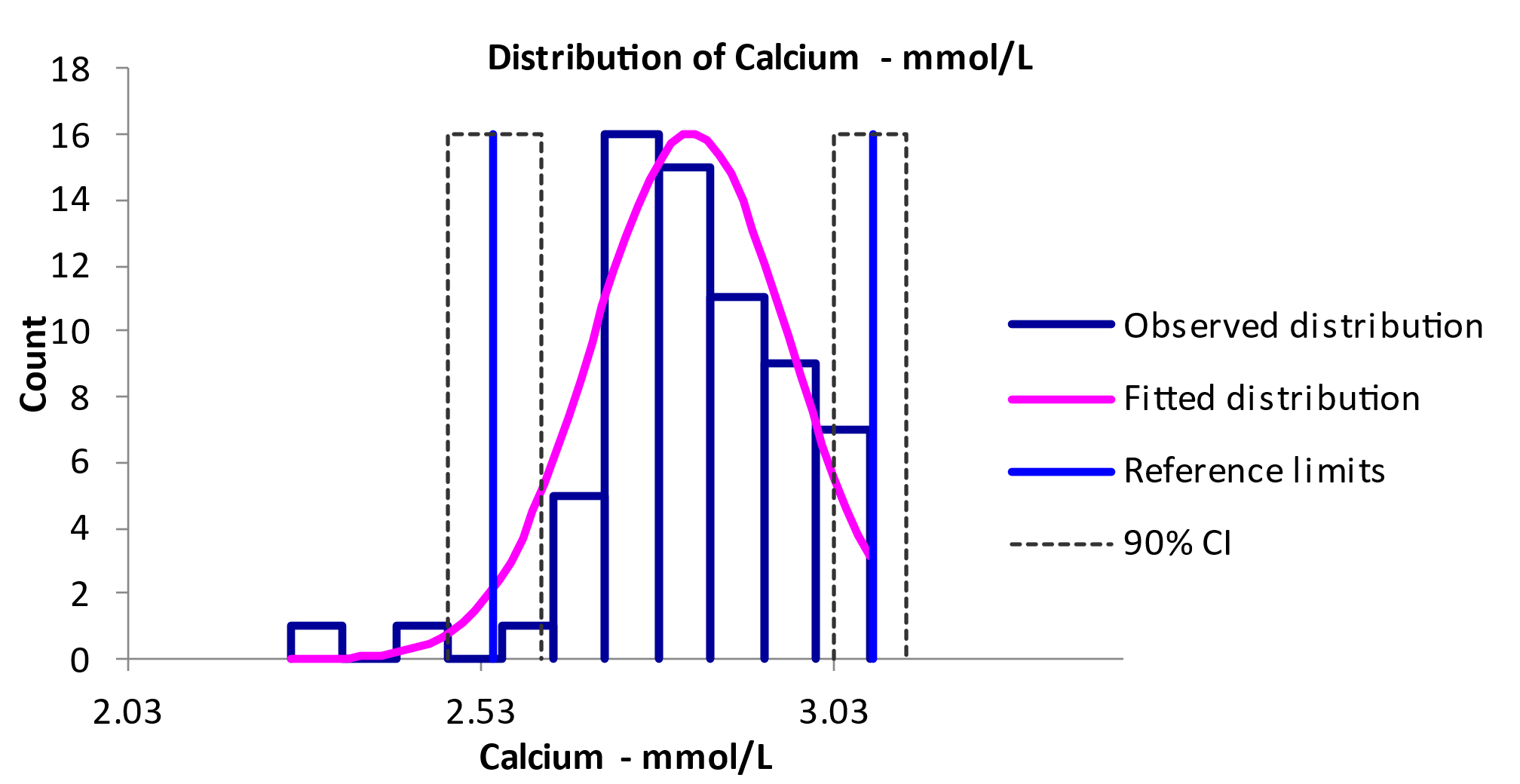

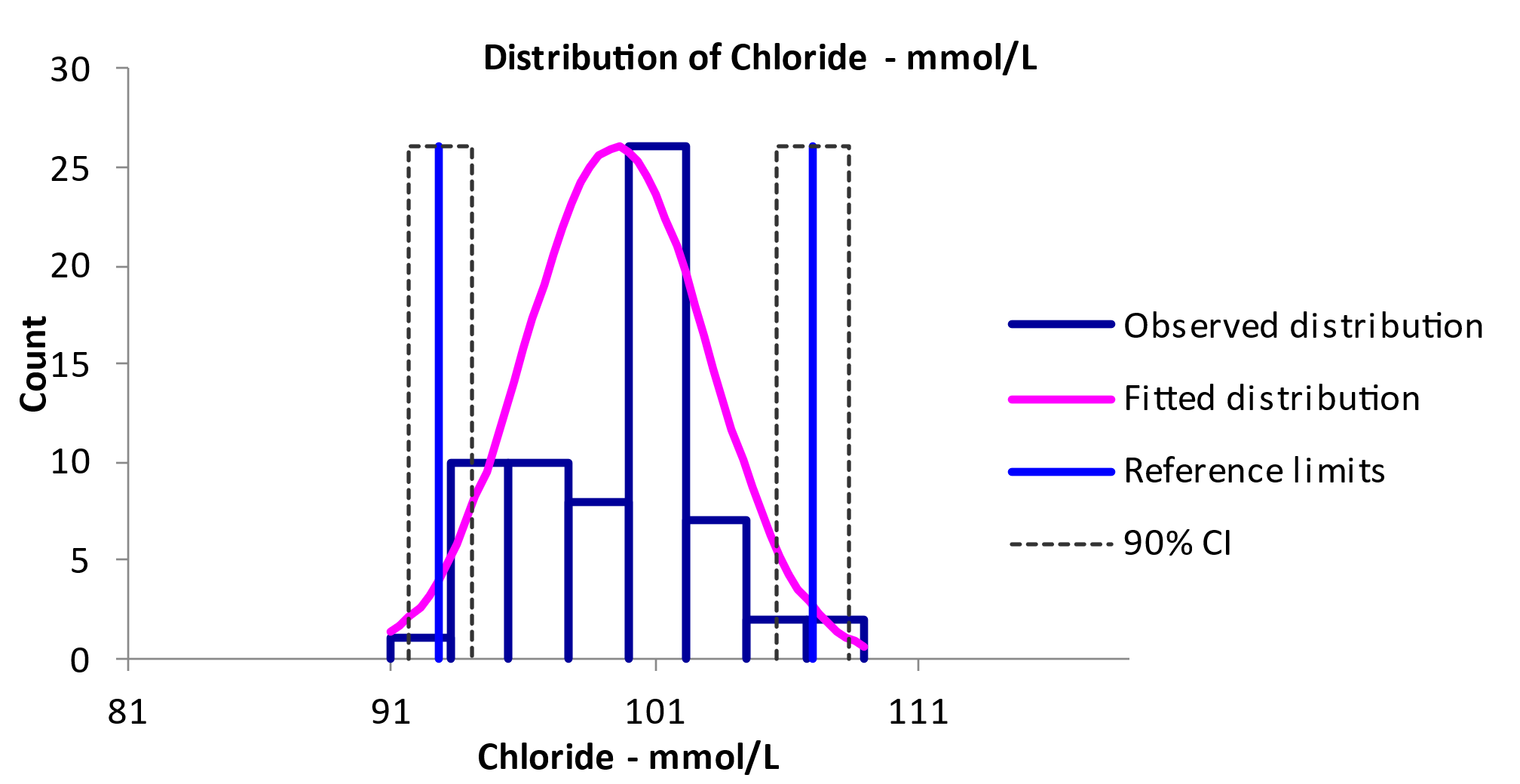


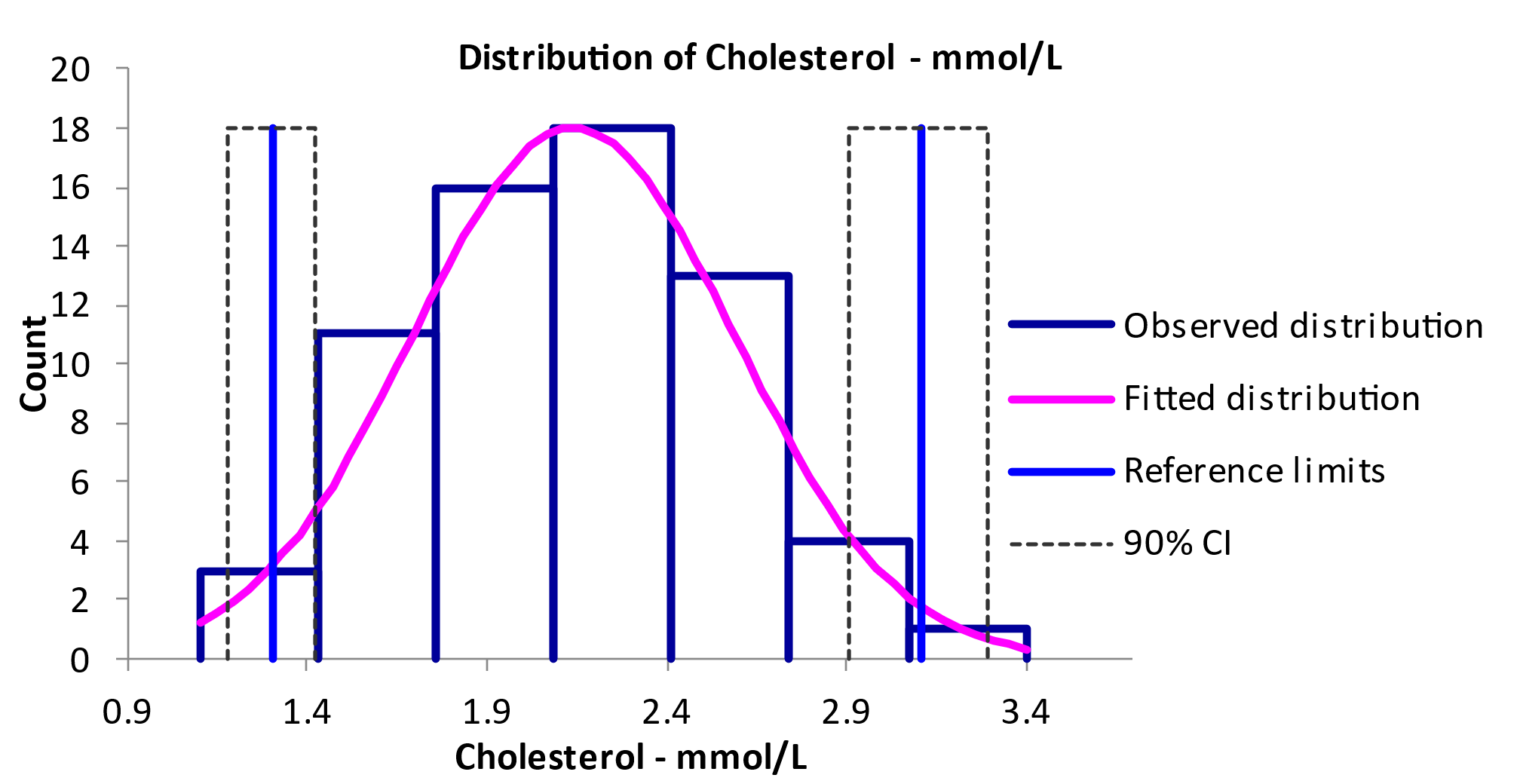

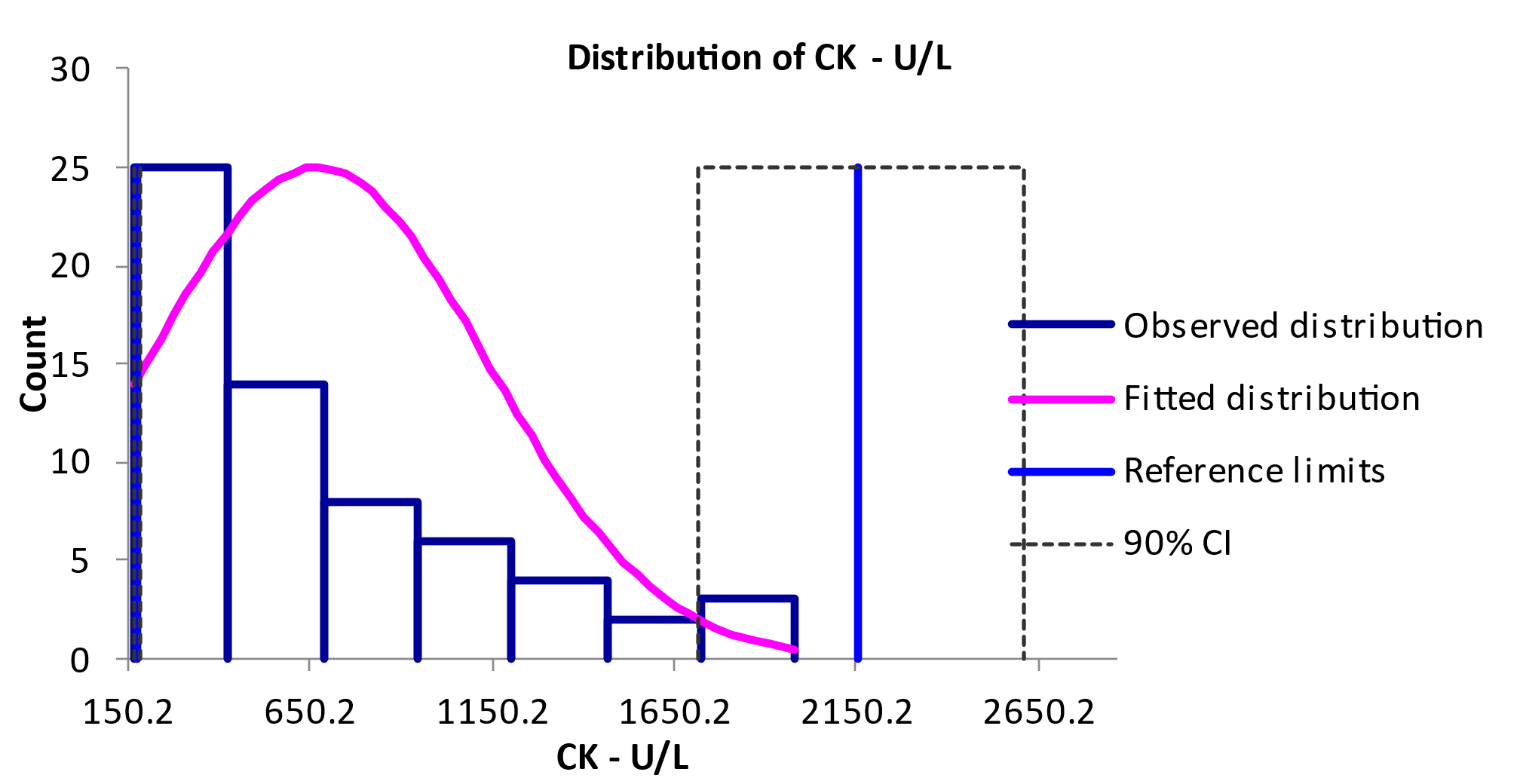


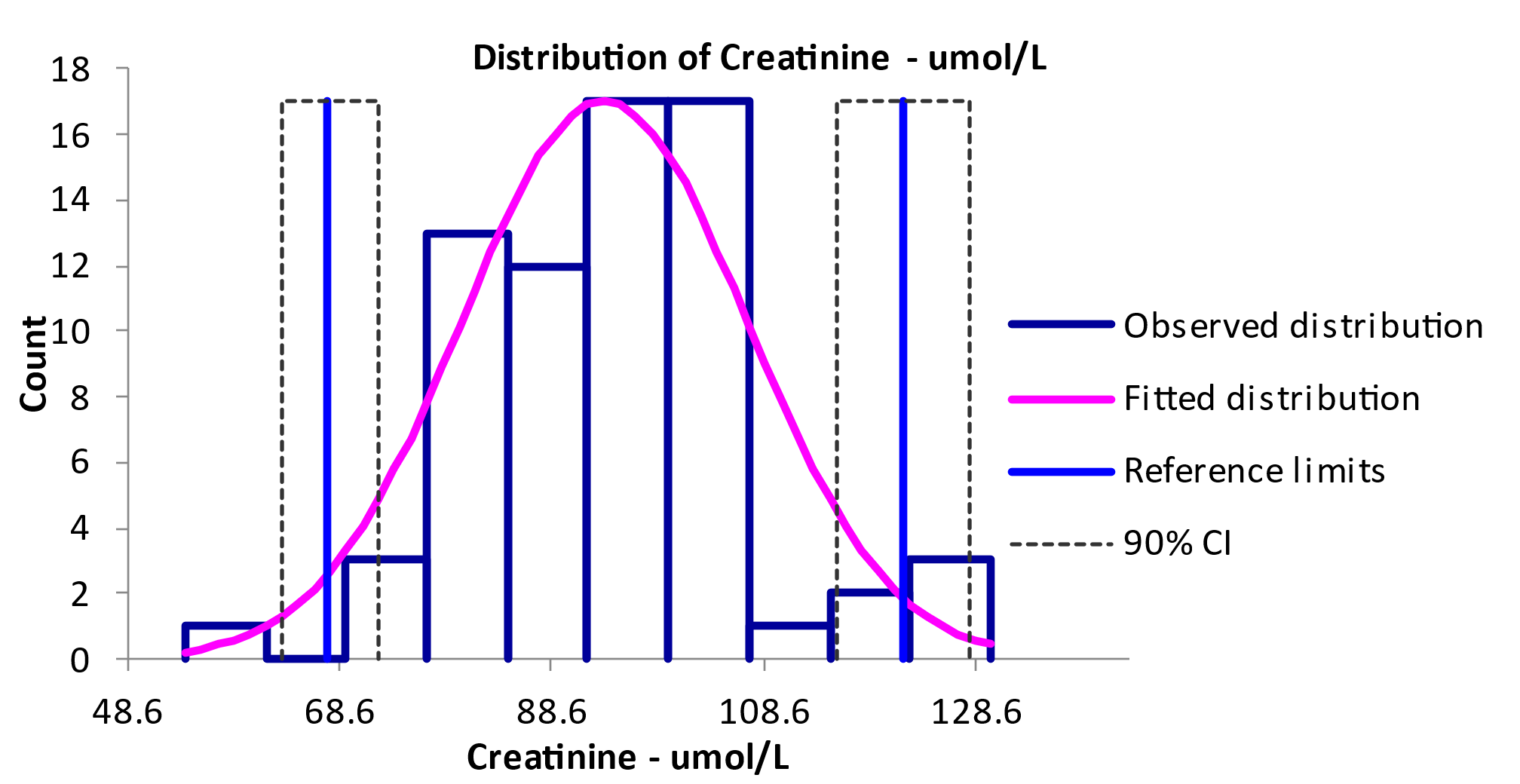

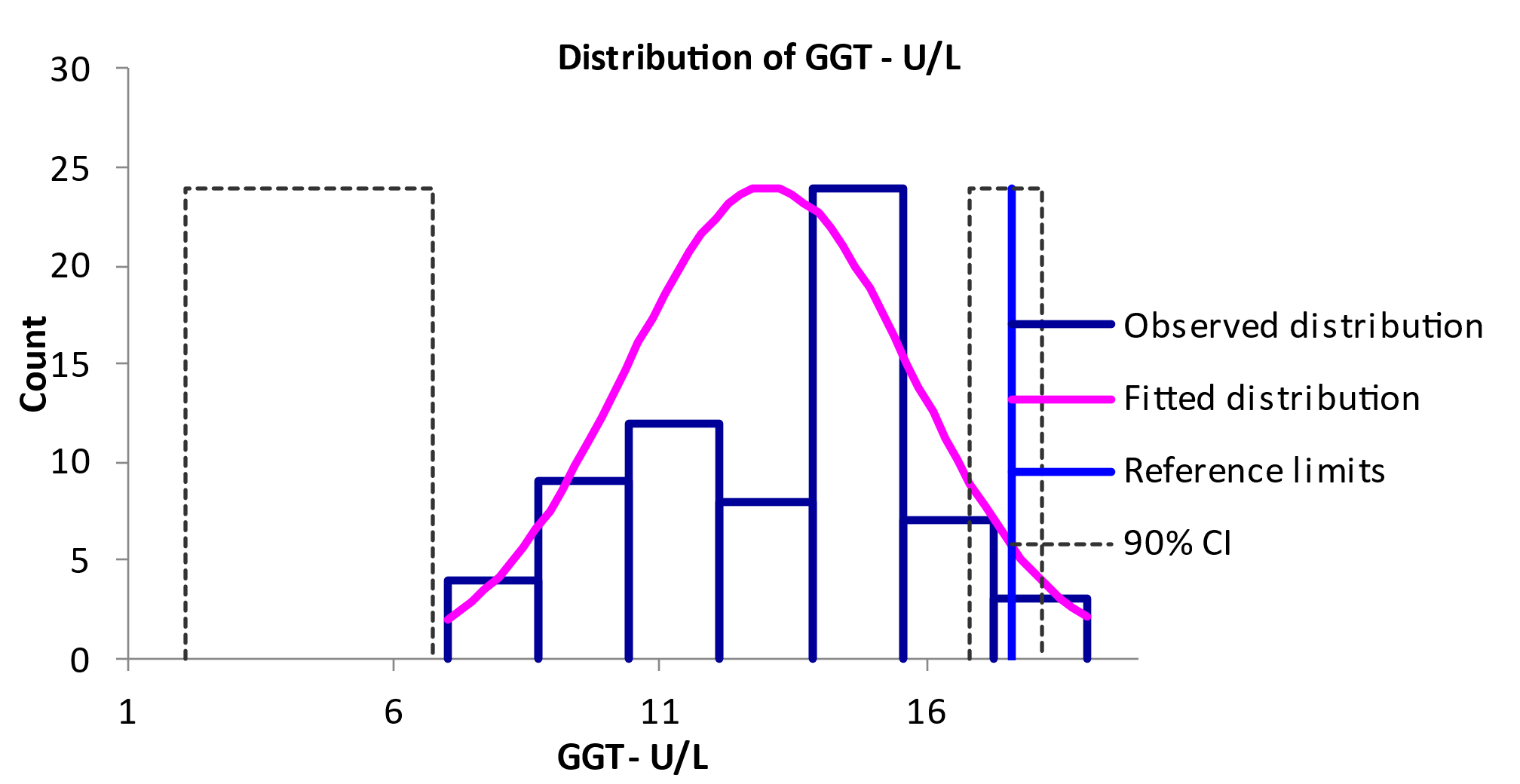


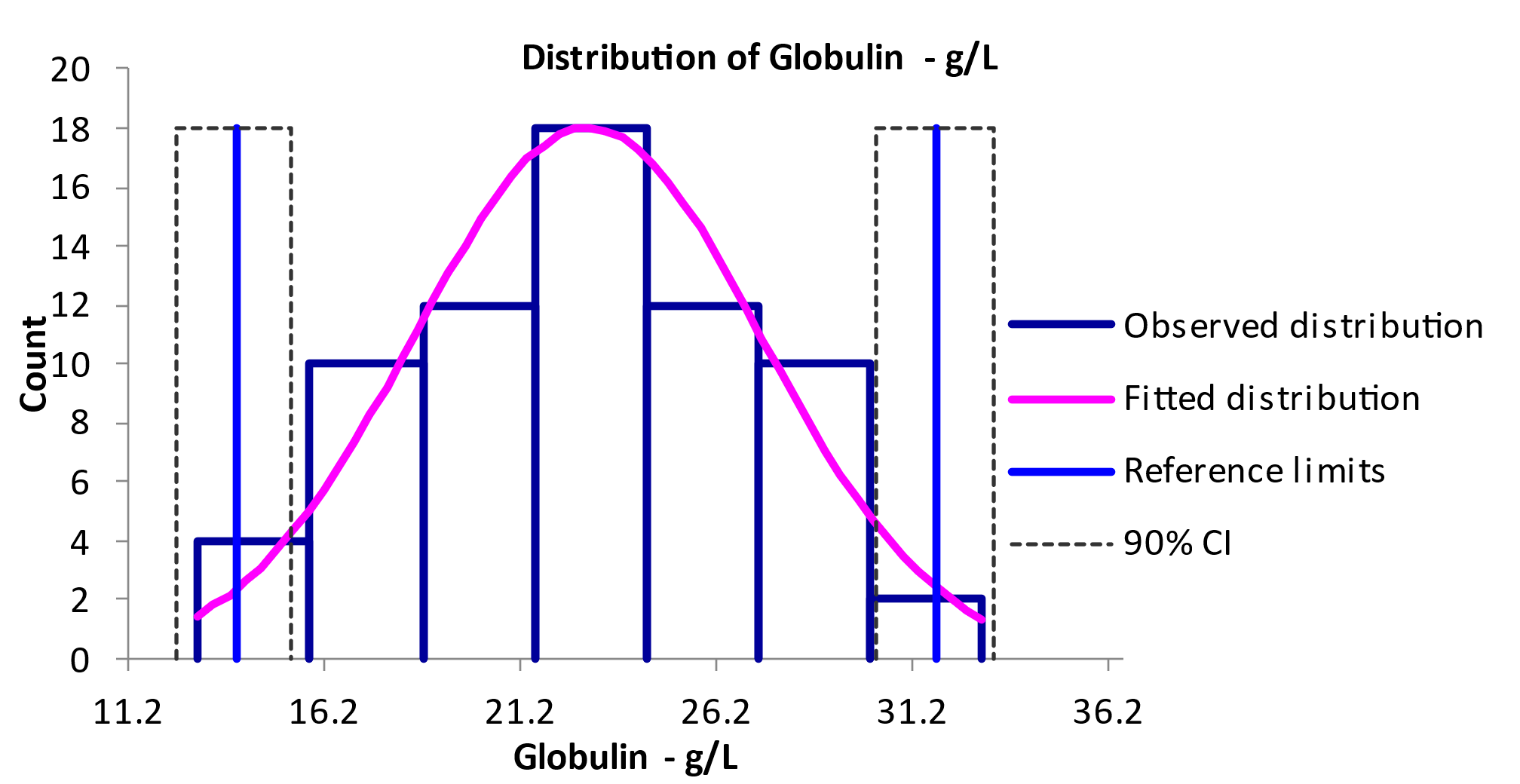

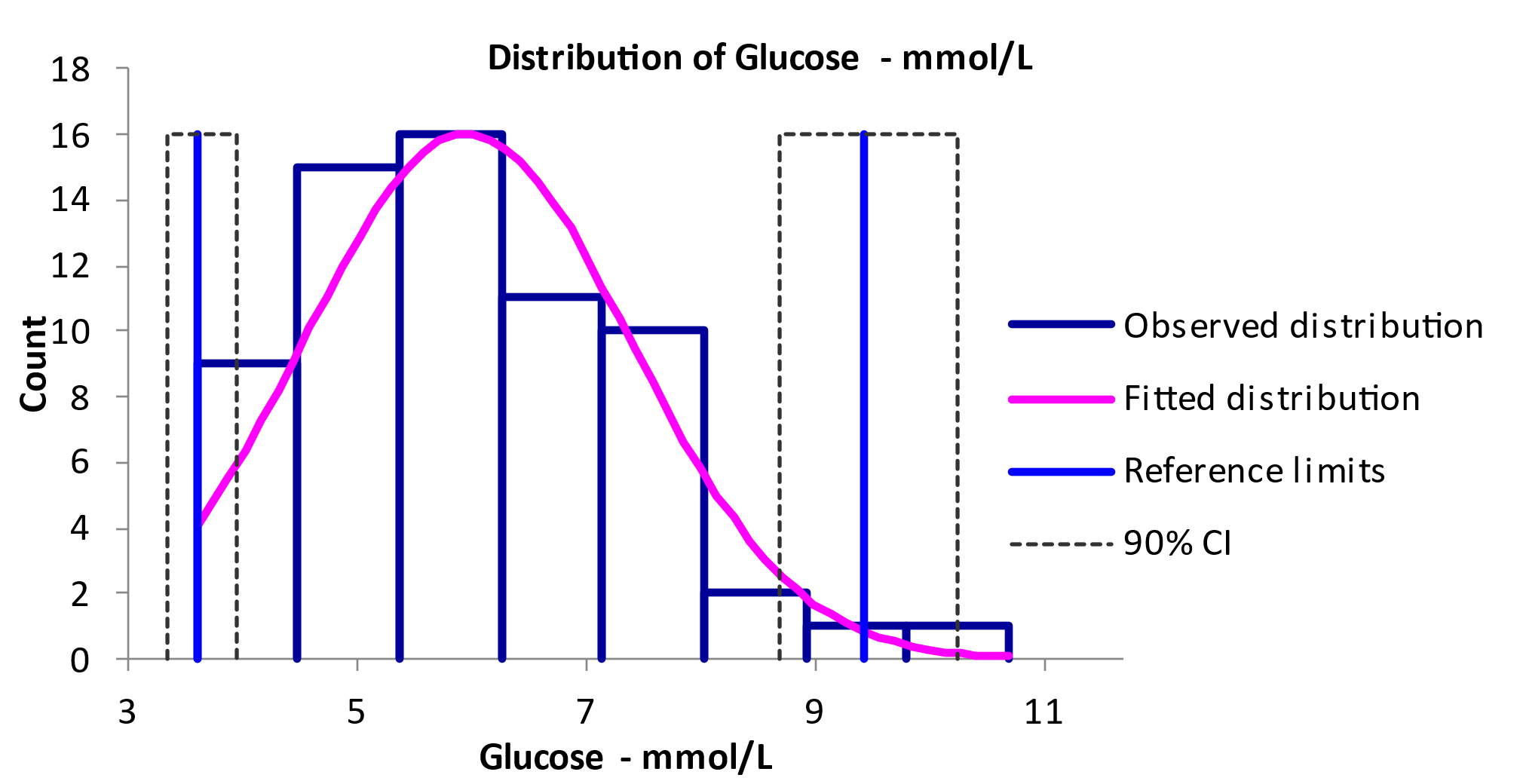


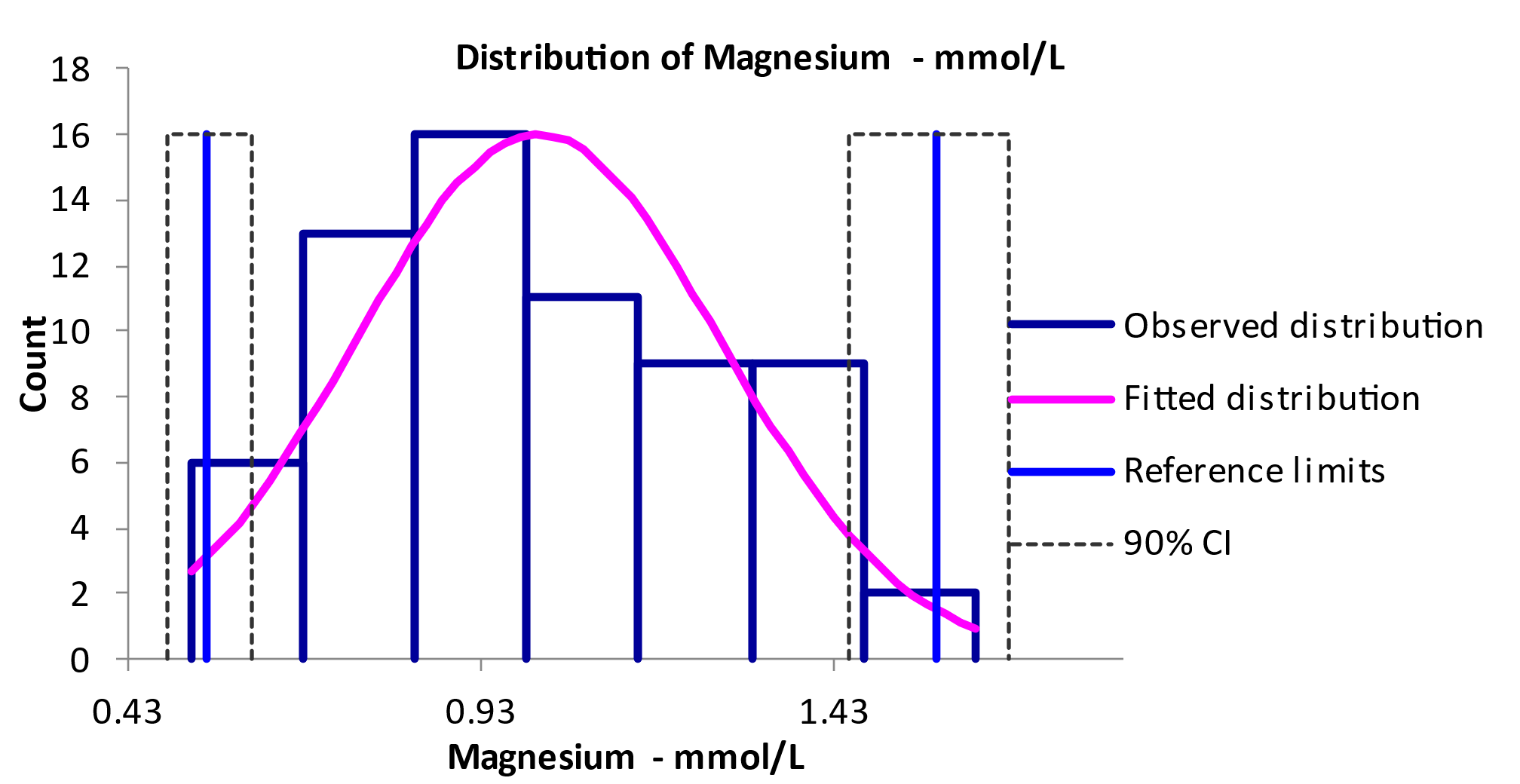

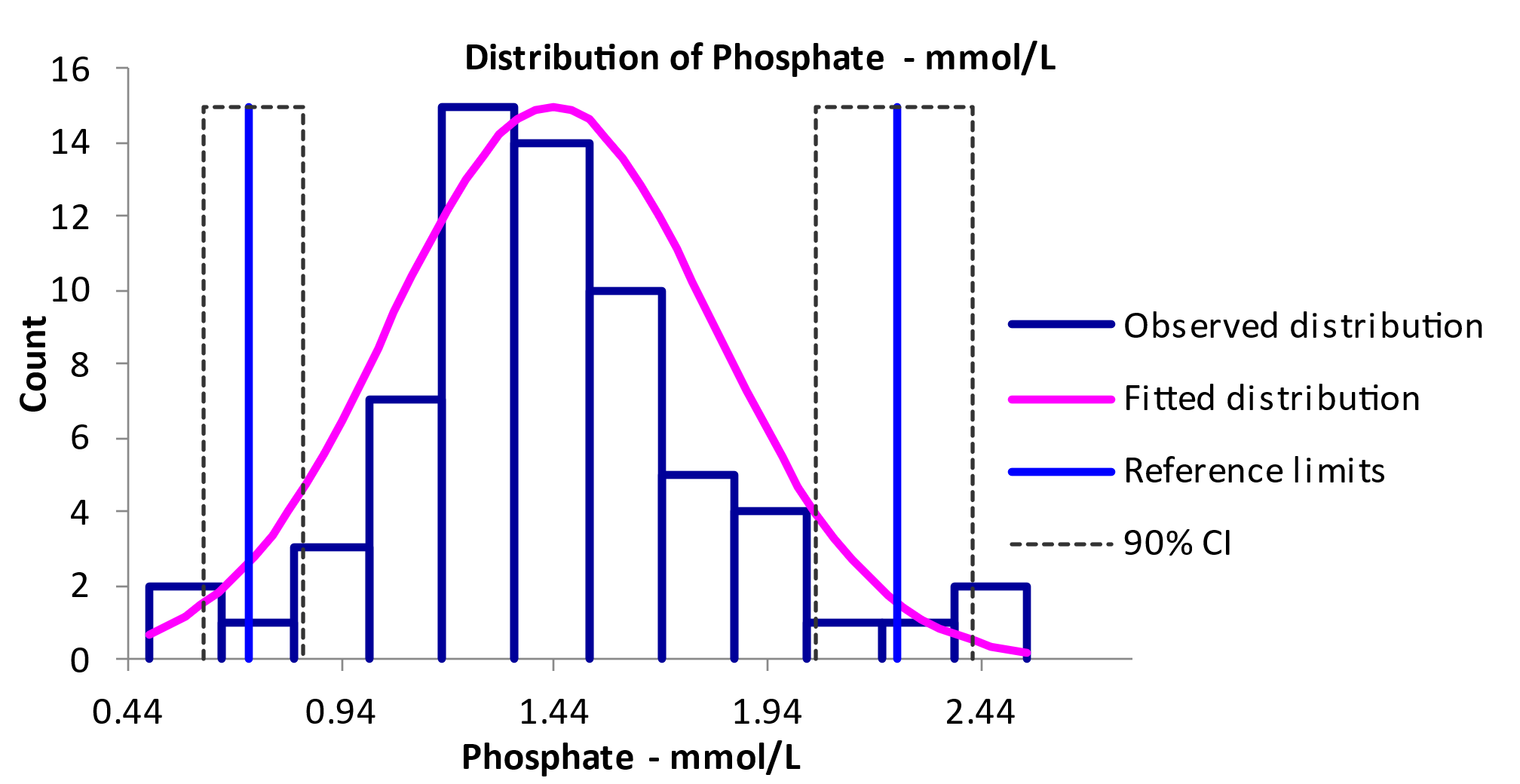


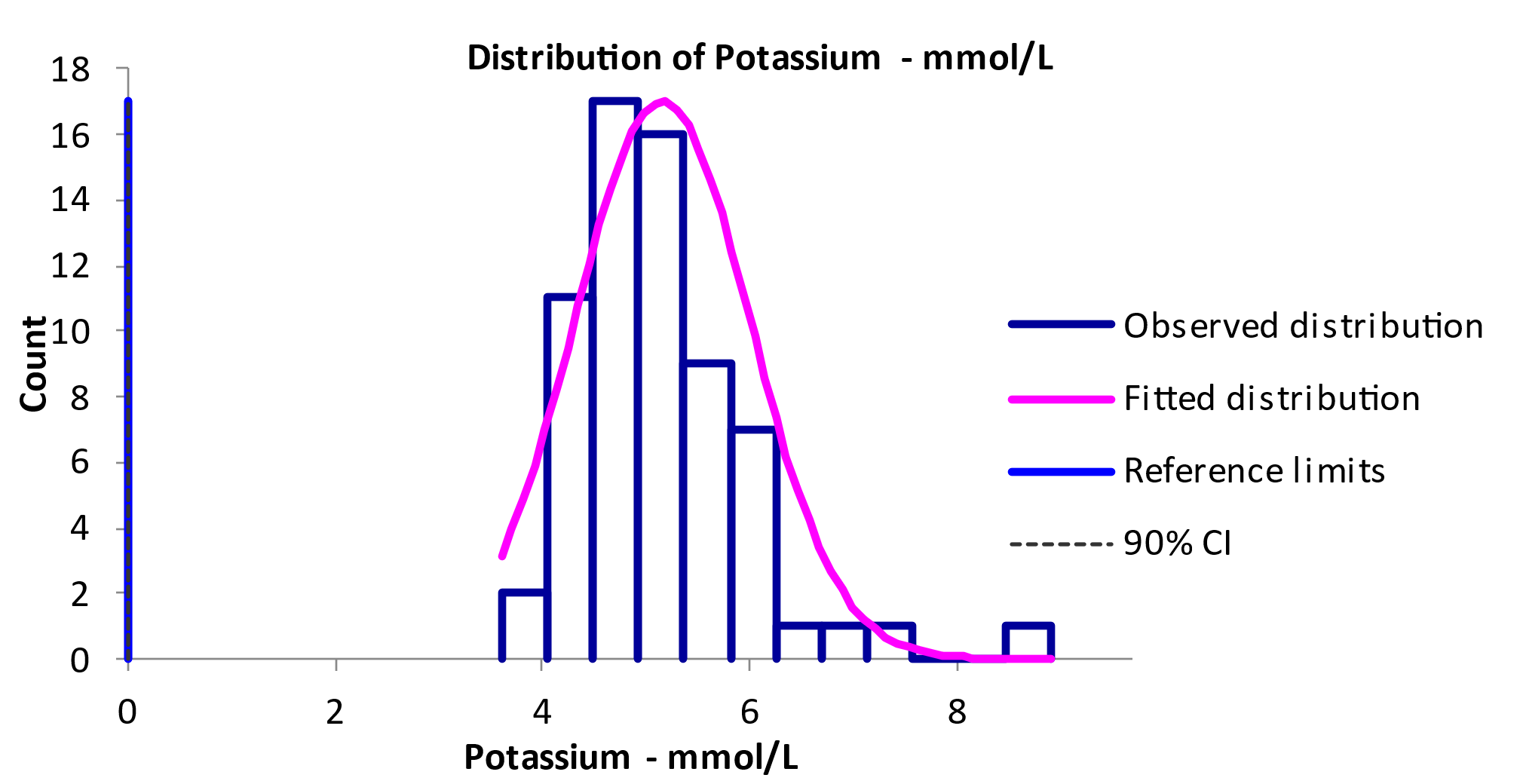

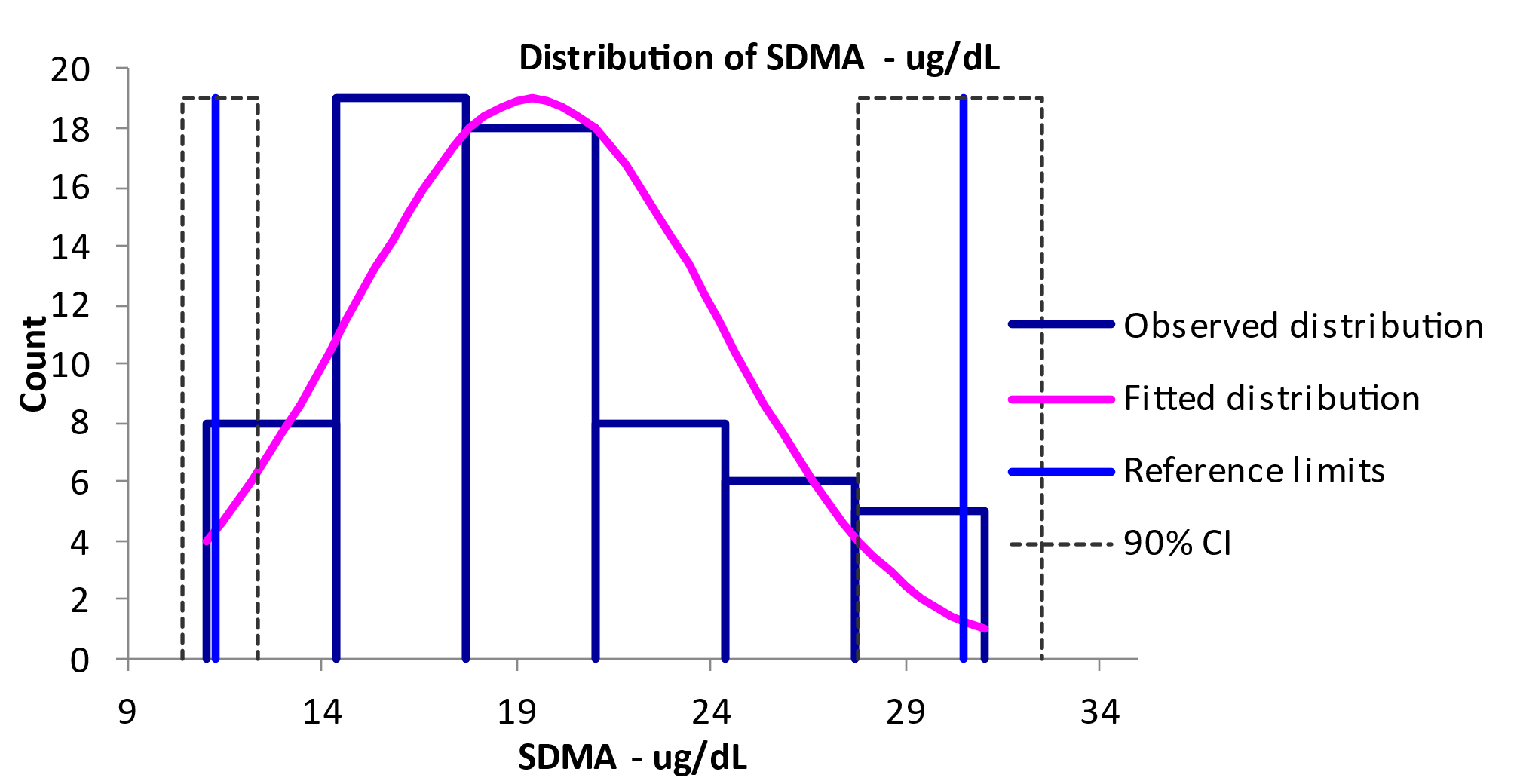


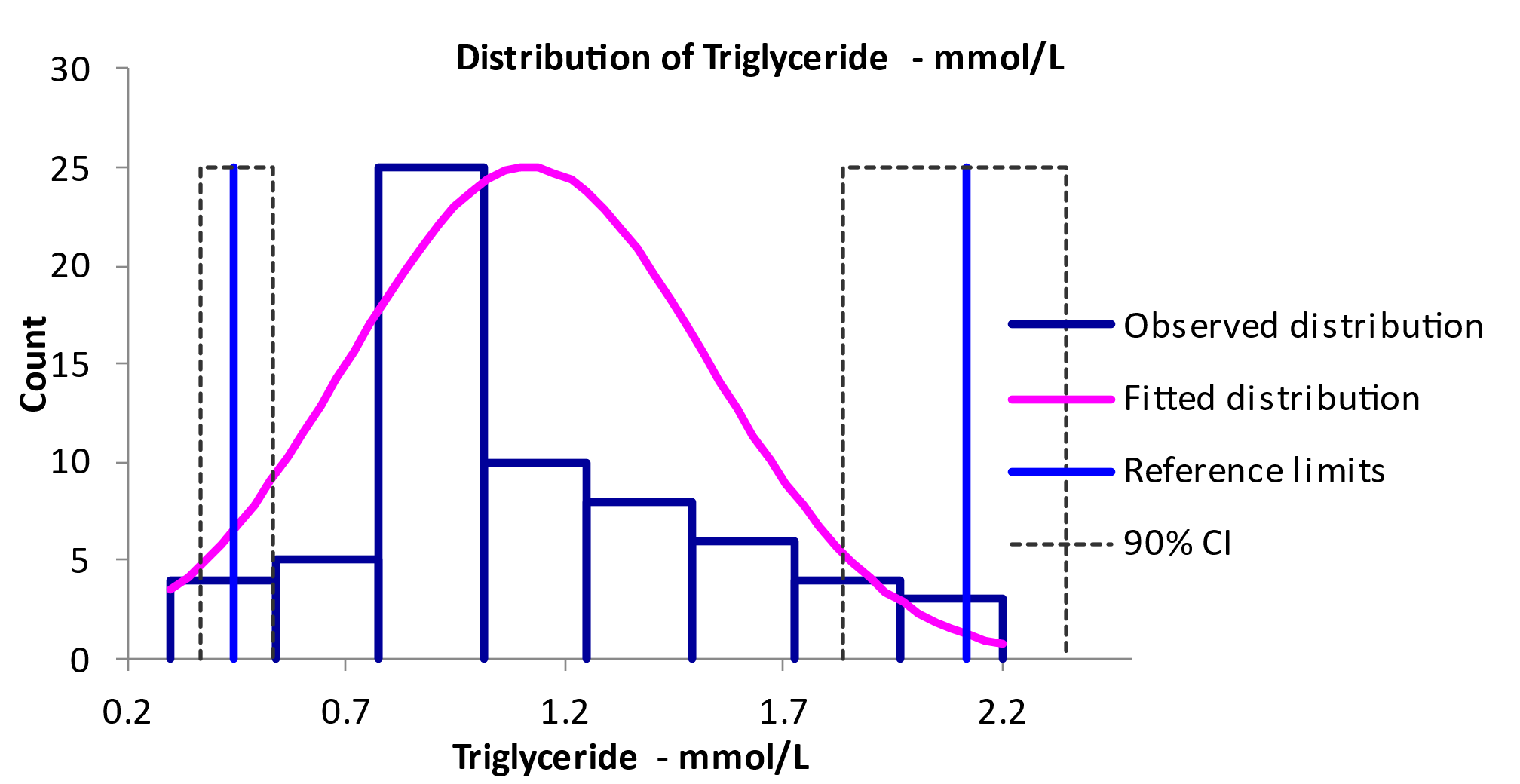

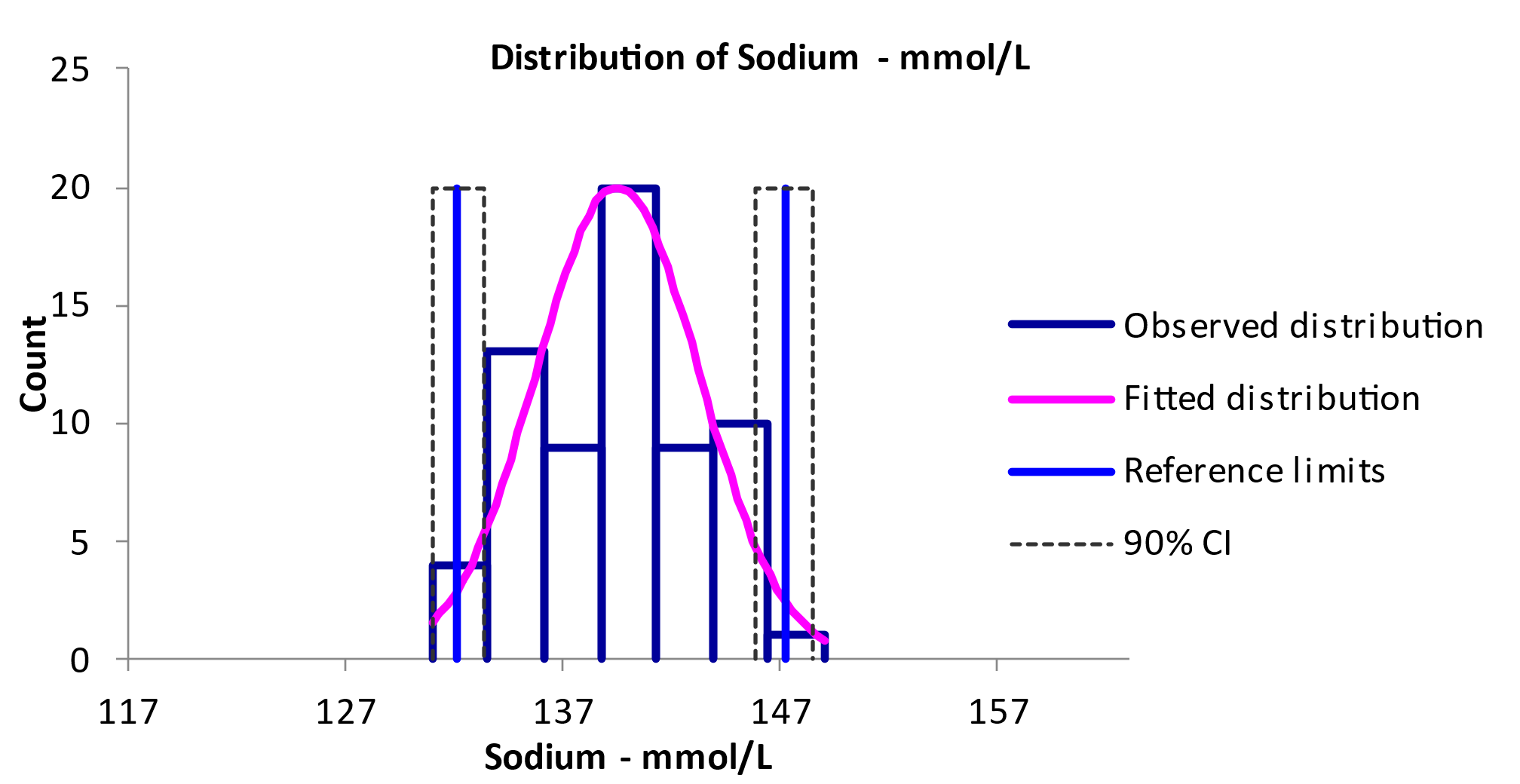


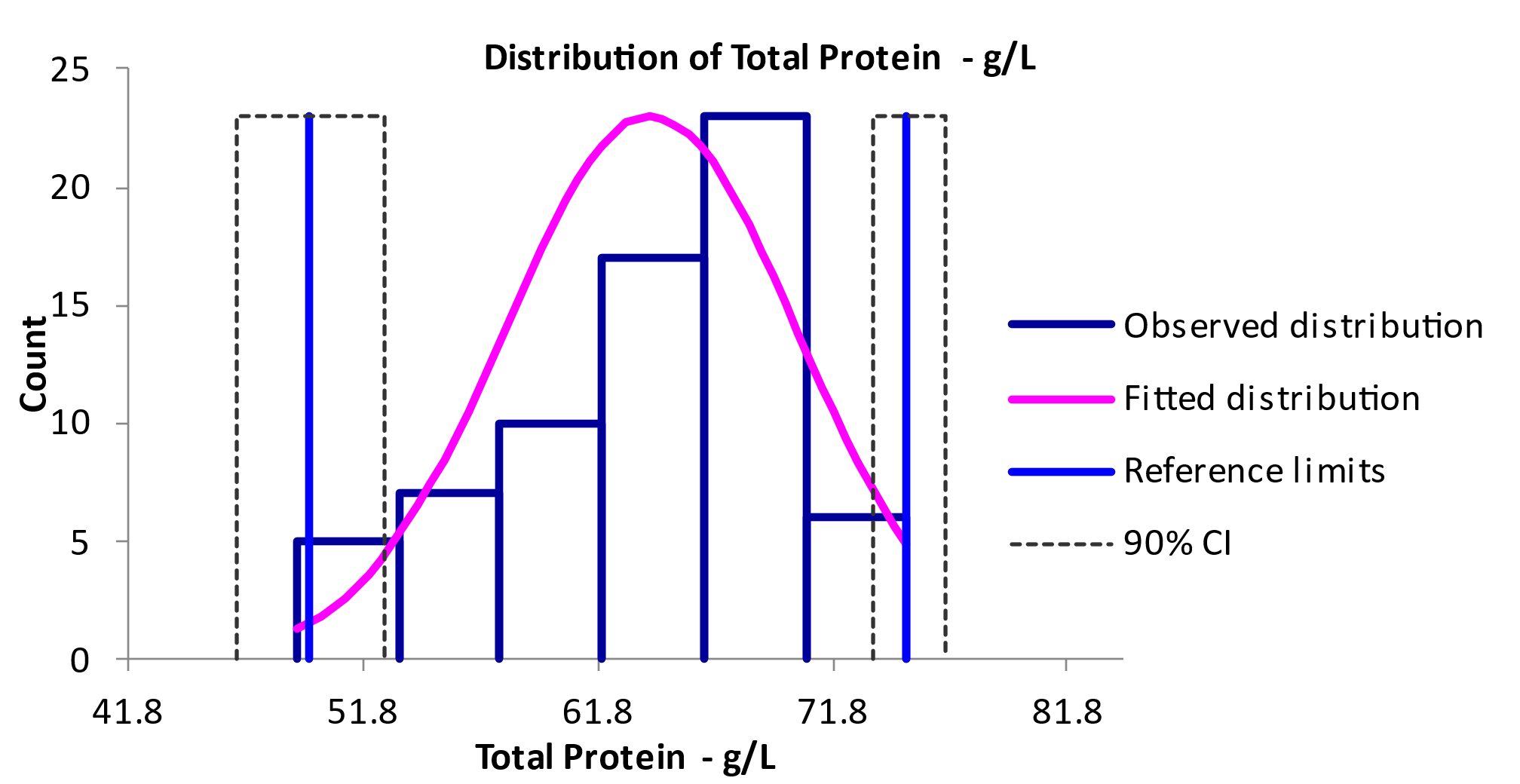

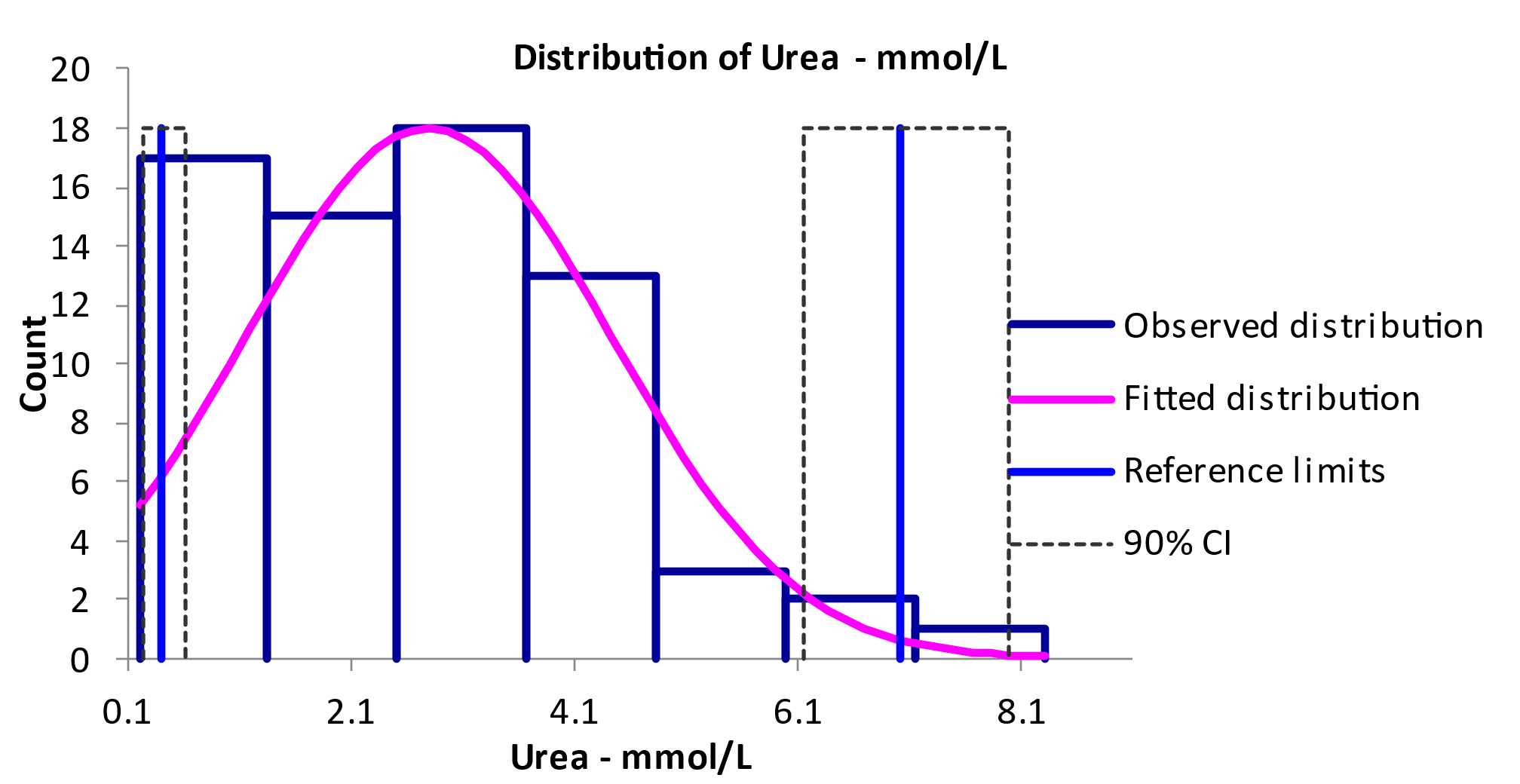

Supplement: Supplementary file 4 — Figure S2. Histograms of analyte distribution for koalas negative for koala retrovirus (KoRV) and Chlamydia pecorum from Reference Value Advisor software. [file VCP-54-300-s001.docx]
