# Supplementary material for: Biochemical Reference Intervals of Free‐Ranging Koalas ( Phascolarctos cinereus ) in South Australia
Source: Vet Clin Pathol. 2025 Jul 2;54(3):300–8. doi: 10.1111/vcp.70024 (PMC12444011; doi:10.1111/vcp.70024)
Supplement: Supplementary file 5 — Figure S3. Histograms of analyte distribution of juvenile koalas from Reference Value Advisor software. [file VCP-54-300-s006.docx]

**FIGURE S3.** Histograms of analyte distribution of juvenile koalas from Reference Value Advisor software.
