# Supplementary material for: Biochemical Reference Intervals of Free‐Ranging Koalas ( Phascolarctos cinereus ) in South Australia
Source: Vet Clin Pathol. 2025 Jul 2;54(3):300–8. doi: 10.1111/vcp.70024 (PMC12444011; doi:10.1111/vcp.70024)
Supplement: Supplementary file 6 — Table S3. Partitioned biochemical variables on the basis of sex, infection status, and location for those analytes with statistical significance. [file VCP-54-300-s005.docx]

| **Analyte** | **Units** | **Infection**  **status** | **n^a^** | **Mean** | **SD** | **Median** | **Min** | **Max** | **Anderson-**  **Darling test** | **Distrib.^b^** | **Method ^c^** | **LRL of**  **RI** | **URL of RI** | **CI 90% of LRL** | **CI 90% of URL** | **P value** |
| --- | --- | --- | --- | --- | --- | --- | --- | --- | --- | --- | --- | --- | --- | --- | --- | --- |
|  |  |  |  |  |  |  |  |  |  |  |  |  |  |  |  |  |
| **Glucose** | mmol/L | KoRV+ | 81 | 5.87 | 1.46 | 5.8 | 3.4 | 10.7 | 0.110 | G | P | 2.9 | 8.8 | 2.5-3.4 | 8.3-9.2 |  |
|  |  | KoRV- | 111 | 5.52 | 1.44 | 5.4 | 2.3 | 10.7 | 0.204 | G | P | 2.6 | 8.4 | 2.3-3.0 | 8.0-8.8 | 0.026 |
| **GGT** | U/L | Chlam+ | 18 | 8.7 | 2.3 | 8.5 | 6 | 14 |  |  |  |  |  |  |  |  |
|  |  | Chlam- | 118 | 12.7 | 2.8 | 13.0 | 7 | 19 | 0.001 | NG | NP | 7.0 | 18.0 | 7-8 | 17-19 | 0.002 |

**^a^** Number of koala samples analysed for each analyte varies due to either sample volume available or outlier exclusion.

^b^Distribution. G= Gaussian, NG= Non-Gaussian.

^c^Statistical method for establishing RI: P, parametric; NP, nonparametric; R, robust; T, transformed.

| **Analyte** | **Units** | | **Sex** | | **n^a^** | | **Mean** | | **SD** | | **Median** | | **Min** | | **Max** | | **Anderson-**  **Darling test** | | **Dist.^b^** | **Method^c^** | | | | **LRL of**  **RI** | | | **URL of RI** | | | **CI 90% of LRL** | | | **CI 90% of URL** | | | | **P value^d^** | |  |  |
| --- | --- | --- | --- | --- | --- | --- | --- | --- | --- | --- | --- | --- | --- | --- | --- | --- | --- | --- | --- | --- | --- | --- | --- | --- | --- | --- | --- | --- | --- | --- | --- | --- | --- | --- | --- | --- | --- | --- | --- | --- |
|  |  |  |  | |  |  |  | |  | |  | |  | |  | |  | |  |  | | | |  |  |  |  | | |  |  |  |  |  |  |  |  | |  |  |
| **Bicarbonate** | mmol/L | | Female | | 68 | | 20 | | 3 | | 20 | | 12 | | 26 | | 0.068 | | G | P | | | | 15 | | | 26 | | | 14-16 | | | 25-27 | | | | 0.043 | |  |  |
|  |  | | Male | | 35 | | 21 | | 3 | | 21 | | 15 | | 27 | | 0.659 | | G | P | | | | 15 | | | 27 | | | 14-17 | | | 26-28 | | | |  |  |  |  |
| **Magnesium** | mmol/L | | Female | | 68 | | 1.03 | | 0.15 | | 1.02 | | 0.7 | | 1.43 | | 0.423 | | G | P | | | | 0.74 | | | 1.33 | | | 0.68-0.79 | | | 1.28-1.38 | | | |  | |  |  |
|  |  |  | Male | | 35 | | 1.17 | | 0.15 | | 1.18 | | 0.82 | | 1.48 | | 0.779 | | G | P | | | | 0.85 | | | 1.49 | | | 0.78-0.92 | | | 1.41-1.56 | | | | 0.002 | |  |  |
| **GGT** | U/L | | Female | | 68 | | 12 | | 3 | | 12 | | 6 | | 17 | | 0.046 | | NG | NP | | | | 6 | | | 17 | | | 6-7 | | | 16-17 | | |  | | |  |  |
|  |  |  | Male | | 35 | | 11 | | 2 | | 11 | | 6 | | 15 | | 0.123 | | G | P | | | | 6 | | | 15 | | | 4-7 | | | 14-17 | | | 0.023 | | |  |  |
| **ALP** | U/L | | Female | | 70 | | 139 | | 126 | | 84 | | 17 | | 589 | | 0.000 | | NG | NP | | | | 19 | | | 579 | | | 17-30 | | | 339-589 | | |  | | |  |  |
|  |  |  | Male | | 36 | | 188 | | 157 | | 138 | | 41 | | 625 | | 0.000 | | NG | T, R | | | | 37 | | | 676 | | | 34-44 | | | 448-939 | | | 0.003 | | |  |  |
| **Triglycerides** | | mmol/L | | Female | | 68 | | 1.4 | | 0.5 | | 1.4 | | 0.4 | | 2.5 | | 0.124 | | G | | P | | | | 0.3 | | | 2.5 | | | 0.2-0.5 | | | 2.3-2.6 | | | |  |  |
|  |  |  |  | Male | | 34 | | 1.2 | | 0.4 | | 1.2 | | 0.5 | | 2.0 | | 0.784 | | G | | P | | | | 0.4 | | | 1.9 | | | 0.3-0.6 | | | 1.8-2.1 | | | | 0.039 |  |
| **Albumin** | | g/L | Female | | 72 | | 39 | | 2.7 | | 40 | | 32 | | 45 | | 0.042 | | NG | | | | P | 33 | | | 45 | | | 32-34 | | | 43-45 | | |  | | | | |
|  |  |  | Male | | 38 | | 37 | | 3.9 | | 39 | | 26 | | 42 | | 0.000 | | NG | | | | T, R | 23 | | | 43 | | | ND-31 | | | 42-43 | | | 0.005 | | | | |

**^a^** Number of koala samples analysed for each analyte varies due to either sample volume available or outlier exclusion.

^b^Distribution. G= Gaussian, NG= Non-Gaussian.

^c^Statistical method for establishing RI: P, parametric; NP, nonparametric; R, robust; T, transformed.

^d^P-value result derived from Mount Lofty Ranges analysis of male and female koala values only.

| **Analyte** | | | **Units** | | | | | | | **Location** | | | | | **n^a^** | | | | **Mean** | | | **SD** | | | **Median** | | | | **Min** | | | | | **Max** | | | | | **Anderson-**  **Darling**  **test** | | | | | **Dist^b^** | | | | | | **Method^c^** | | | | | | **LRL**  **of RI** | | | | **URL**  **of RI** | | | | | **CI 90%**  **of LRL** | | | | **CI 90%**  **of URL** | | | **P value** | | | | | | |  |  |  |
| --- | --- | --- | --- | --- | --- | --- | --- | --- | --- | --- | --- | --- | --- | --- | --- | --- | --- | --- | --- | --- | --- | --- | --- | --- | --- | --- | --- | --- | --- | --- | --- | --- | --- | --- | --- | --- | --- | --- | --- | --- | --- | --- | --- | --- | --- | --- | --- | --- | --- | --- | --- | --- | --- | --- | --- | --- | --- | --- | --- | --- | --- | --- | --- | --- | --- | --- | --- | --- | --- | --- | --- | --- | --- | --- | --- | --- | --- | --- | --- | --- | --- |
|  |  |  |  |  |  |  |  |  |  |  | | | | |  |  |  |  |  | | |  | | |  | | | |  | |  | | | | | | |  | | | | |  | | | | | |  | | | | | |  | | | | | |  | | | | |  | | | |  | | | | |  | |  |  |  |  |  |
| **Sodium** | | | | | mmol/L | | | | | MLR | | | | | | | 103 | | | | | 143 | | 4 | | | | 142 | | | 134 | | | | 153 | | | 0.008 | | | | | | | NG | | | | | | NP | | | | | | 136 | | | | | 151 | | | | | 134-137 | | | | 149-153 | | | | |  | | | | |  |
|  |  |  |  |  |  |  |  |  |  | KI | | | | | | | 92 | | | | | 139 | | 3 | | | | 139 | | | 131 | | | | 147 | | | 0.175 | | | | | | | G | | | | | | P | | | | | | 133 | | | | | 146 | | | | | 132-133 | | | | 145-147 | | | | | <0.001 | | | | |  |
| **Potassium** | | | | | mmol/L | | | | | MLR | | | | | | | 103 | | | | | 4.78 | | 0.64 | | | | 4.70 | | | 3.6 | | | | 7.4 | | | 0.054 | | | | | | | G | | | | | | NP | | | | | | 3.7 | | | | | 6.12 | | | | | 3.6-3.9 | | | | 5.94-7.4 | | | | |  | | | | |  |
|  |  |  |  |  |  |  |  |  |  | KI | | | | | | | 92 | | | | | 5.23 | | 0.91 | | | | 5.1 | | | 3.6 | | | | 8.9 | | | 0.000 | | | | | | | NG | | | | | | NP | | | | | | 4.0 | | | | | 7.6 | | | | | 3.6-4.2 | | | | 7.0-8.9 | | | | | <0.001 | | | | |  |
| **Chloride** | | | | | mmol/L | | | | | MLR | | | | | | | 103 | | | | | 102 | | 4 | | | | 102 | | | 92 | | | | 113 | | | 0.166 | | | | | | | G | | | | | NP | | | | | | 95 | | | | | | 109 | | | | | 92-95 | | | | 108-113 | | | | |  | | | | |  |
|  |  |  |  |  |  |  |  |  |  | KI | | | | | | | 92 | | | | | 99 | | 3 | | | | 100 | | | 89 | | | | 107 | | | 0.019 | | | | | | | NG | | | | | NP | | | | | | 92 | | | | | | 106 | | | | | 89-94 | | | | 104-107 | | | | | <0.001 | | | | |  |
| **Bicarbonate** | | | | | mmol/L | | | | | MLR | | | | | | | 104 | | | | | 20.43 | | 2.8 | | | | 20 | | | 12 | | | | 27 | | | 0.044 | | | | | | | NG | | | | | NP | | | | | | 13.6 | | | | | | 26.0 | | | | | 12-16 | | | | 25-27 | | | | |  | | | | |  |
|  |  |  |  |  |  |  |  |  |  | KI | | | | | | | 92 | | | | | 17.6 | | 2.8 | | | | 17 | | | 12 | | | | 25 | | | 0.004 | | | | | | | NG | | | | | NP | | | | | | 13.3 | | | | | | 24.0 | | | | | 12-14 | | | | 22.7-25 | | | | | <0.001 | | | | |  |
| **Magnesium** | | | | | mmol/L | | | | | MLR | | | | | | | 103 | | | | | 1.08 | | 0.16 | | | | 1.08 | | | 0.7 | | | | 1.48 | | | 0.843 | | | | | | | G | | | | | NP | | | | | | 0.76 | | | | | | 1.42 | | | | | 0.7-0.82 | | | | 1.36-1.48 | | | | |  | | | | |  |
|  |  |  |  |  |  |  |  |  |  | KI | | | | | | | 93 | | | | | 1.01 | | 0.26 | | | | 0.98 | | | 0.52 | | | | 1.63 | | | 0.190 | | | | | | | G | | | | | P | | | | | | 0.49 | | | | | | 1.56 | | | | | 0.41-0.56 | | | | 1.45-1.61 | | | | | 0.018 | | | | |  |
| **Calcium** | | | | | mmol/L | | | | | MLR | | | | | | | 103 | | | | | 2.74 | | 0.28 | | | | 2.68 | | | 2.34 | | | | 4.19 | | | 0.000 | | | | | | | NG | | | | | NP | | | | | | 2.39 | | | | | | 3.72 | | | | | 2.34-2.44 | | | | 3.11-4.19 | | | | |  | | | | |  |
|  |  |  |  |  |  |  |  |  |  | KI | | | | | | | 92 | | | | | 2.81 | | 0.15 | | | | 2.82 | | | 2.24 | | | | 3.08 | | | 0.001 | | | | | | | NG | | | | | NP | | | | | | 2.30 | | | | | | 3.04 | | | | | 2.24-2.59 | | | | 3.03-3.08 | | | | | <0.001 | | | | |  |
| **Phosphate** | | | | mmol/L | | | | MLR | | | | 102 | | | | | 1.13 | | | | 0.31 | | | 1.14 | | | 0.29 | | | | | | 2.07 | | | | 0.244 | | | | G | | | | NP | | | | | | | 0.56 | | | | | 1.78 | | | | | 0.29-0.67 | | | | | | 1.71-2.07 | | | |  | | | |  |  |  |  |  |  |
|  |  |  |  |  |  |  |  | KI | | | | 92 | | | | | 1.54 | | | | 0.38 | | | 1.5 | | | 0.92 | | | | | | 2.75 | | | | 0.001 | | | | NG | | | | NP | | | | | | | 0.98 | | | | | 2.55 | | | | | 0.92-1.03 | | | | | | 2.36-2.75 | | | | <0.001 | | | |  |  |  |  |  |  |
| **Urea** | | | | mmol/L | | | | MLR | | | | 113 | | | | | 2.9 | | | | 1.2 | | | 2.8 | | | 0.3 | | | | | | 6.3 | | | | 0.569 | | | | G | | | | NP | | | | | | | 0.6 | | | | | 5.4 | | | | | 0.3-0.9 | | | | | | 4.9-6.3 | | | |  | | | |  |  |  |  |  |  |
|  |  |  |  |  |  |  |  | KI | | | | 92 | | | | | 3.3 | | | | 1.9 | | | 3.1 | | | 0.2 | | | | | | 9.2 | | | | 0.017 | | | | NG | | | | NP | | | | | | | 0.6 | | | | | 8.0 | | | | | 0.2-0.9 | | | | | | 6.5-9.2 | | | | 0.033 | | | |  |  |  |  |  |  |
| **SDMA** | | | | µg/dL | | | | MLR | | | | 102 | | | | | 14.0 | | | 3.5 | | | 13 | | | 9 | | | | | | 26 | | | | 0.000 | | | | NG | | | | NP | | | | | | | 9.0 | | | | | 25.4 | | | | | 9-10 | | | | | | 21-26 | | | |  | | | |  |  |  |  |  |  |  |
|  |  |  |  |  |  |  |  | KI | | | | 91 | | | | | 19.8 | | | 4.3 | | | 19 | | | 11 | | | | | | 31 | | | | 0.009 | | | | NG | | | | NP | | | | | | | 12.3 | | | | | 28.7 | | | | | 11-13 | | | | | | 28-31 | | | | <0.001 | | | |  |  |  |  |  |  |  |
| **AST** | | | | U/L | | | | MLR | | | | 107 | | | | | 21 | | | 10 | | | 18 | | | 10 | | | | | | 63 | | | | 0.000 | | | | NG | | | | NP | | | | | | | 10 | | | | | 54 | | | | | 10-12 | | | | | | 44-63 | | | |  | | | |  |  |  |  |  |  |  |
|  |  |  |  |  |  |  |  | KI | | | | 89 | | | | | 28 | | | 13 | | | 23 | | | 9 | | | | | | 68 | | | | 0.000 | | | | NG | | | | NP | | | | | | | 10 | | | | | 63 | | | | | 9-12 | | | | | | 53-68 | | | | <0.001 | | | |  |  |  |  |  |  |  |
| **CK** | | | | | U/L | | | | MLR | | | | 104 | | | | | 293 | | | 190 | | | 236 | | | 99 | | | | | | 1227 | | | | 0.000 | | | | NG | | | | NP | | | | | | | 113 | | | | | 873 | | | | | 99-127 | | | | | | 699-1227 | | | |  | | | |  |  |  |  |  |  |
|  |  |  |  |  |  |  |  |  | KI | | | | 83 | | | | | 771 | | | 493 | | | 608 | | | 190 | | | | | | 2148 | | | | 0.000 | | | | NG | | | | NP | | | | | | | 200 | | | | | 2093 | | | | | 190-260 | | | | | | 1951-2148 | | | | <0.001 | | | |  |  |  |  |  |  |
| **GGT** | | | | | U/L | | | | MLR | | | | 104 | | | | | 11 | | | 3 | | | 11 | | | 6 | | | | | | 17 | | | | 0.023 | | | | NG | | | | NP | | | | | | | 6 | | | | | 16 | | | | | 6-7 | | | | | | 15-17 | | | |  | | | |  |  |  |  |  |  |
|  |  |  |  |  |  |  |  |  | KI | | | | 93 | | | | | 13 | | | 3 | | | 14 | | | 7 | | | | | | 19 | | | | 0.004 | | | | NG | | | | NP | | | | | | | 8 | | | | | 19 | | | | | 7-8 | | | | | | 17-19 | | | | <0.001 | | | |  |  |  |  |  |  |
| **ALP** | | | | U/L | | | | MLR | | | | 106 | | | | | 147 | | | 134 | | | 89.5 | | | 17 | | | | | | 625 | | | | 0.000 | | | | NG | | | | NP | | | | | | | 26 | | | | | 586 | | | | | 17-36 | | | | | | 441-625 | | | |  | | | |  |  |  |  |  |  |  |
|  |  |  |  |  |  |  |  | KI | | | | 93 | | | | | 206 | | | 143 | | | 149 | | | 30 | | | | | | 598 | | | | 0.000 | | | | NG | | | | NP | | | | | | | 49 | | | | | 562 | | | | | 30-56 | | | | | | 480-598 | | | | <0.001 | | | |  |  |  |  |  |  |  |
| **ALT** | | | | U/L | | | | MLR | | | | 107 | | | | | 8 | | | 3 | | | 7 | | | 3 | | | | | | 20 | | | | 0.000 | | | | NG | | | | NP | | | | | | | 4 | | | | | 18 | | | | | 3-5 | | | | | | 14-20 | | | |  | | | |  |  |  |  |  |  |  |
|  |  |  |  |  |  |  |  | KI | | | | 93 | | | | | 13.5 | | | 8 | | | 11 | | | 5 | | | | | | 63 | | | | 0.000 | | | | NG | | | | NP | | | | | | | 6 | | | | | 42 | | | | | 5-6 | | | | | | 24-63 | | | | <0.001 | | | |  |  |  |  |  |  |  |
| **Glucose** | | | | | | | mmol/L | | | | | MLR | | | | | 102 | | | | 5.2 | | | 1.4 | | | 5.1 | | | | 2.3 | | | | | | 10.7 | | | | 0.119 | | | | | | G | | | | | NP | | | | | | | 2.3 | | | | | 8.3 | | | | 2.3-3.0 | | | | 7.5-10.7 | | |  | | | | |  |  |
|  |  |  |  |  |  |  |  |  |  |  |  | KI | | | | | 92 | | | | 6.1 | | | 1.4 | | | 5.9 | | | | 3.6 | | | | | | 10.7 | | | | 0.066 | | | | | | G | | | | | P | | | | | | | 3.4 | | | | | 8.9 | | | | 3.0-3.8 | | | | 8.5-9.3 | | | <0.001 | | | | |  |  |
| **Triglycerides** | | | | | | | mmol/L | | | | | MLR | | | | | 102 | | | | 1.3 | | | 0.5 | | | 1.3 | | | | 0.4 | | | | | | 2.5 | | | | 0.117 | | | | | | G | | | | | NP | | | | | | | 0.5 | | | | | 2.4 | | | | 0.4-0.5 | | | | 2.3-2.5 | | |  | | | | |  |  |
|  |  |  |  |  |  |  |  |  |  |  |  | KI | | | | | 93 | | | | 1.1 | | | 0.5 | | | 1.0 | | | | 0.3 | | | | | | 2.7 | | | | 0.000 | | | | | | NG | | | | | NP | | | | | | | 0.4 | | | | | 2.6 | | | | 0.3-0.5 | | | | 2.0-2.7 | | | 0.014 | | | | |  |  |
| **Cholesterol** | | | | | | | | mmol/L | | | | | MLR | | | | | 103 | | | | 1.8 | | | 0.3 | | | 1.7 | | | | 1.2 | | | | | | 2.7 | | | | 0.010 | | | | | | NG | | | | | NP | | | | | | | 1.2 | | | | | 2.5 | | | | 1.2-1.3 | | | | 2.4-2.7 | | |  | | | | |  |
|  |  |  |  |  |  |  |  |  |  |  |  |  | KI | | | | | 93 | | | | 2.2 | | | 0.5 | | | 2.2 | | | | 1.1 | | | | | | 3.7 | | | | 0.067 | | | | | | G | | | | | P | | | | | | | 1.2 | | | | | 3.2 | | | | 1.1-1.4 | | | | 3.1-3.4 | | | <0.001 | | | | |  |
| **Total Protein** | | | | | | | | g/L | | | | | MLR | | | | | 112 | | | | 59 | | | 5 | | | 59 | | | | 46 | | | | | | 68 | | | | 0.026 | | | | | | NG | | | | | NP | | | | | | | 47 | | | | | 68 | | | | 46-49 | | | | 66-68 | | |  | | | | |  |
|  |  |  |  |  |  |  |  |  |  |  |  |  | KI | | | | | 93 | | | | 65 | | | 6 | | | 66 | | | | 51 | | | | | | 75 | | | | 0.004 | | | | | | NG | | | | | NP | | | | | | | 51 | | | | | 75 | | | | 51-54 | | | | 73-75 | | | <0.001 | | | | |  |
| **Albumin** | | | | | | | | | g/L | | | | | MLR | | | | | 110 | | | | 39 | | | 3 | | | 39 | | | | 26 | | | | | | 45 | | | | 0.000 | | | | | | NG | | | | | NP | | | | | | | 29 | | | | | 44 | | | | 26-33 | | | | 43-45 | | |  | | | | |
|  |  |  |  |  |  |  |  |  |  |  |  |  |  | KI | | | | | 93 | | | | 42 | | | 3 | | | 42 | | | | 33 | | | | | | 47 | | | | 0.002 | | | | | | NG | | | | | NP | | | | | | | 36 | | | | | 46 | | | | 33-36 | | | | 46-47 | | | <0.001 | | | | |
| **Globulin^d^** | | | | | | | | | g/L | | | | | MLR | | | | | 110 | | | | 20 | | | 3 | | | 20 | | | | 13 | | | | | | 28 | | | | 0.109 | | | | | | G | | | | | NP | | | | | | | 13 | | | | | 26 | | | | 13-15 | | | | 25-28 | | |  | | | | |
|  |  |  |  |  |  |  |  |  |  |  |  |  |  | KI | | | | | 93 | | | | 23 | | | 4 | | | 24 | | | | 13 | | | | | | 33 | | | | 0.496 | | | | | | G | | | | | P | | | | | | | 15 | | | | | 32 | | | | 13-16 | | | | 30-33 | | | <0.001 | | | | |

**^a^** Number of koala samples analysed for each analyte varies due to either sample volume available or outlier exclusion.

^b^Distribution. G= Gaussian, NG= Non-Gaussian.

^c^Statistical method for establishing RI: P, parametric; NP, nonparametric; R, robust; T, transformed.

**^d^**Calculated analytes (others measured). Globulin=Total protein-albumin.
